# Toolkit of Approaches To Support Target-Focused Drug Discovery for *Plasmodium falciparum* Lysyl tRNA Synthetase

**DOI:** 10.1021/acsinfecdis.2c00364

**Published:** 2022-08-29

**Authors:** Rachel Milne, Natalie Wiedemar, Victoriano Corpas-Lopez, Eoin Moynihan, Richard J. Wall, Alice Dawson, David A. Robinson, Sharon M. Shepherd, Robert J. Smith, Irene Hallyburton, John M. Post, Karen Dowers, Leah S. Torrie, Ian H. Gilbert, Beatriz Baragaña, Stephen Patterson, Susan Wyllie

**Affiliations:** Wellcome Centre for Anti-Infectives Research, School of Life Sciences, University of Dundee, Dow Street, Dundee DD1 5EH, U.K.

**Keywords:** *Plasmodium*, lysyl tRNA synthetase, thermal proteome profiling (TPP), isothermal TPP, chemical pulldown, antimalarial drug discovery

## Abstract

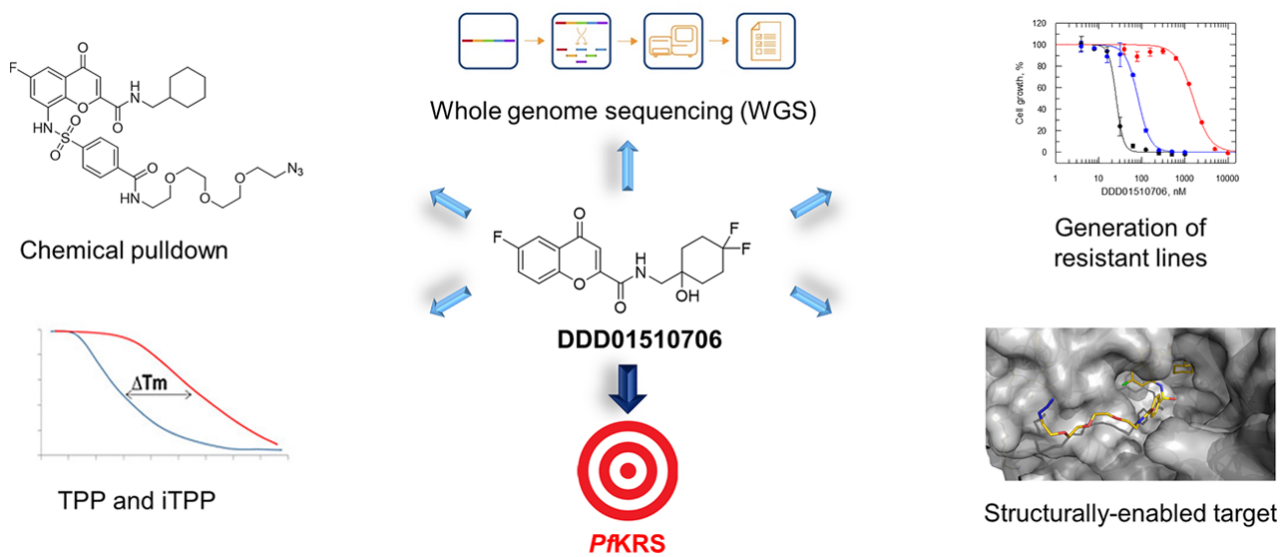

There is a pressing need for new medicines to prevent and treat malaria. Most antimalarial drug discovery is reliant upon phenotypic screening. However, with the development of improved target validation strategies, target-focused approaches are now being utilized. Here, we describe the development of a toolkit to support the therapeutic exploitation of a promising target, lysyl tRNA synthetase (*Pf*KRS). The toolkit includes resistant mutants to probe resistance mechanisms and on-target engagement for specific chemotypes; a hybrid KRS protein capable of producing crystals suitable for ligand soaking, thus providing high-resolution structural information to guide compound optimization; chemical probes to facilitate pulldown studies aimed at revealing the full range of specifically interacting proteins and thermal proteome profiling (TPP); as well as streamlined isothermal TPP methods to provide unbiased confirmation of on-target engagement within a biologically relevant milieu. This combination of tools and methodologies acts as a template for the development of future target-enabling packages.

Malaria is a life-threatening disease that results in more than 600,000 deaths every year, with children under the age of five among the most vulnerable to infection (World Malaria Report 2019, World Health Organization). The disease results from infection with protozoan parasites from the genus *Plasmodium*, transmitted through the bite of the *Anopheles* mosquito. The vast majority of malaria deaths are caused by *P. falciparum* and *P. vivax*. Current antimalarial control is heavily reliant upon a range of artemisinin-based combination therapies (ACTs). However, the efficacy of these front-line therapies is now being threatened by emerging resistance, with ACT treatment failure rates in some regions of Southeast Asia reaching 50%.^1^ Even more concerning is the increasing incidence of mutations associated with ACT resistance in *P. falciparum* isolates from Uganda^2^ and Rwanda.^3^ Should this trend herald the emergence of wide-spread resistance in sub-Saharan Africa where cases and deaths from *P. falciparum* malaria are extremely high, the consequences could be catastrophic.^4^ In addition, there is a need for new medicines for chemoprotection and prevention of transmission and activity against the hypnozoite stage for the purpose of elimination and eradication.^5^

The development of new antimalarials is complicated by a number of factors. The lifecycle of the *Plasmodium* parasite is complex. Following initial transmission, sporozoites enter dermal blood vessels and travel through the bloodstream to the liver where they invade hepatocytes. Parasites replicate and differentiate within hepatocytes prior to entering the bloodstream, where merozoites invade red blood cells. Intraerythrocytic infection is characterized by repeated rounds of asexual replication (schizogony), resulting in a huge expansion of the parasite population. A small number of blood-stage parasites differentiate into transmissible sexual forms (gametocytes). Control and eradication of malaria will be dependent upon the development of compounds that are active against the majority of parasite lifecycle stages, thus presenting a significant challenge for drug discovery. A further impediment is the dearth of robustly validated drug targets in *Plasmodium*, thus limiting target-focused screening programs.^6^

Protein synthesis has proven an aspect of *Plasmodium* metabolism that is readily exploitable for antimalarial drug discovery. The commonly used malaria prophylactic drug doxycycline is thought to inhibit protein synthesis by binding directly to the 30S subunit of the parasite ribosome. *P. falciparum* translation elongation factor 2, responsible for the GTP-dependent translocation of the ribosome along mRNA, has been identified as the molecular target of DDD107498.^7^ M5717, a quinoline-4-carboxamide, is undergoing human trials to establish its suitability as a component of a single-dose antimalarial combination therapy.^8^

Aminoacyl-tRNA synthetases, which catalyze the aminoacylation of tRNAs with their cognate amino acids, have also shown promise as targets for chemotherapeutic intervention. These enzymes work through a two-step process, initially activating the amino acid through reaction with ATP, with the activated amino acid then transferred to the cognate tRNA. tRNA synthetases have multiple sites to facilitate binding of ATP, the amino acid, tRNA, and in some cases an editing site to cleave incorrectly charged tRNAs. Novel bicyclic azetidines, active in mouse models of malaria, specifically target cytosolic phenylalanyl-tRNA synthetase,^9^ while antimalarials borrelidin and halofuginone inhibit threonyl-tRNA synthetase^10^ and prolyl-tRNA synthetase,^11^ respectively. Most notably, the fungal secondary metabolite cladosporin (Figure 1), a potent inhibitor of parasite growth in blood and liver stages of *Plasmodium* was confirmed to specifically target cytosolic lysyl tRNA synthetase (KRS^12^). Unfortunately, a number of factors including poor bioavailability, metabolic instability, and a lack of chemical tractability make cladosporin itself unsuitable for progression as an antimalarial.^13^ Thus, cytosolic *Pf*KRS has become the focus of a target-based drug discovery program in our laboratory with the aim of identifying novel chemotypes capable of inhibiting this promising molecular target. These studies are ongoing; however, a chromone tool molecule (DDD01510706) that interacts with the ATP binding site of *Pf*KRS has been developed and used to validate this enzyme as a viable drug target in animal models of malaria.^13^

Here, we describe the assembly of a toolkit of approaches to facilitate the development of potent *Pf*KRS inhibitors with therapeutic potential. Specifically, this toolkit is being used to guide the discovery and development of new chemotypes capable of inhibiting *Pf*KRS to provide structural information to guide medicinal chemistry optimization of inhibitors and to confirm that compounds in the optimization phase of the drug discovery process remain on target. Importantly, this toolkit could be deployed to monitor clinical isolates during any subsequent clinical development of *Pf*KRS inhibitors for resistance and to understand how that resistance may arise. We envisage that similar toolkits can be developed to support and accelerate target-based structure-guided drug discovery projects focused on other novel antimalarial targets.

## Results and Discussion

### Generation of Chromone-Resistant *P. falciparum* Cell Lines

**Figure 1:**
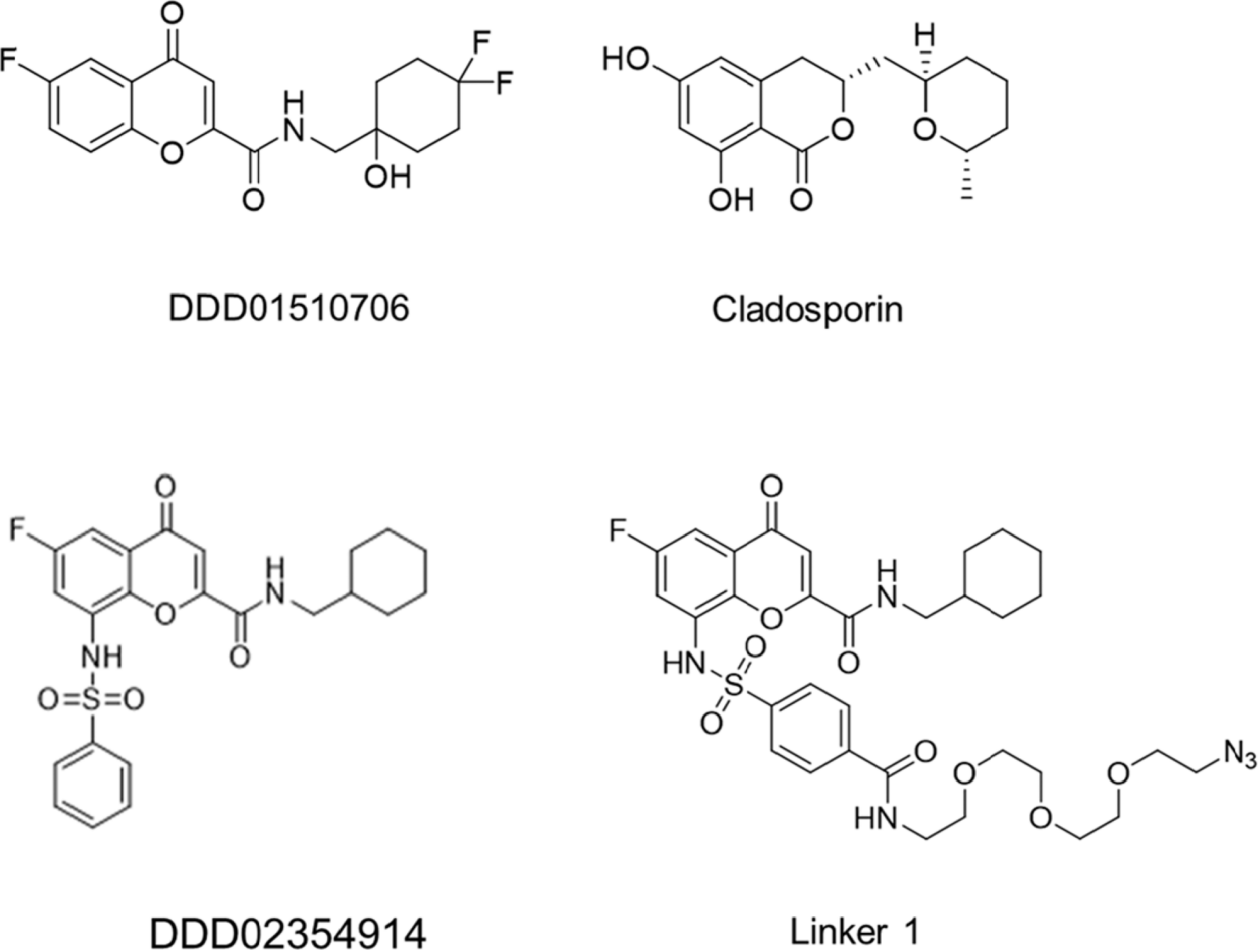
Chemical structures of compounds used in this study.

**Figure 2:**
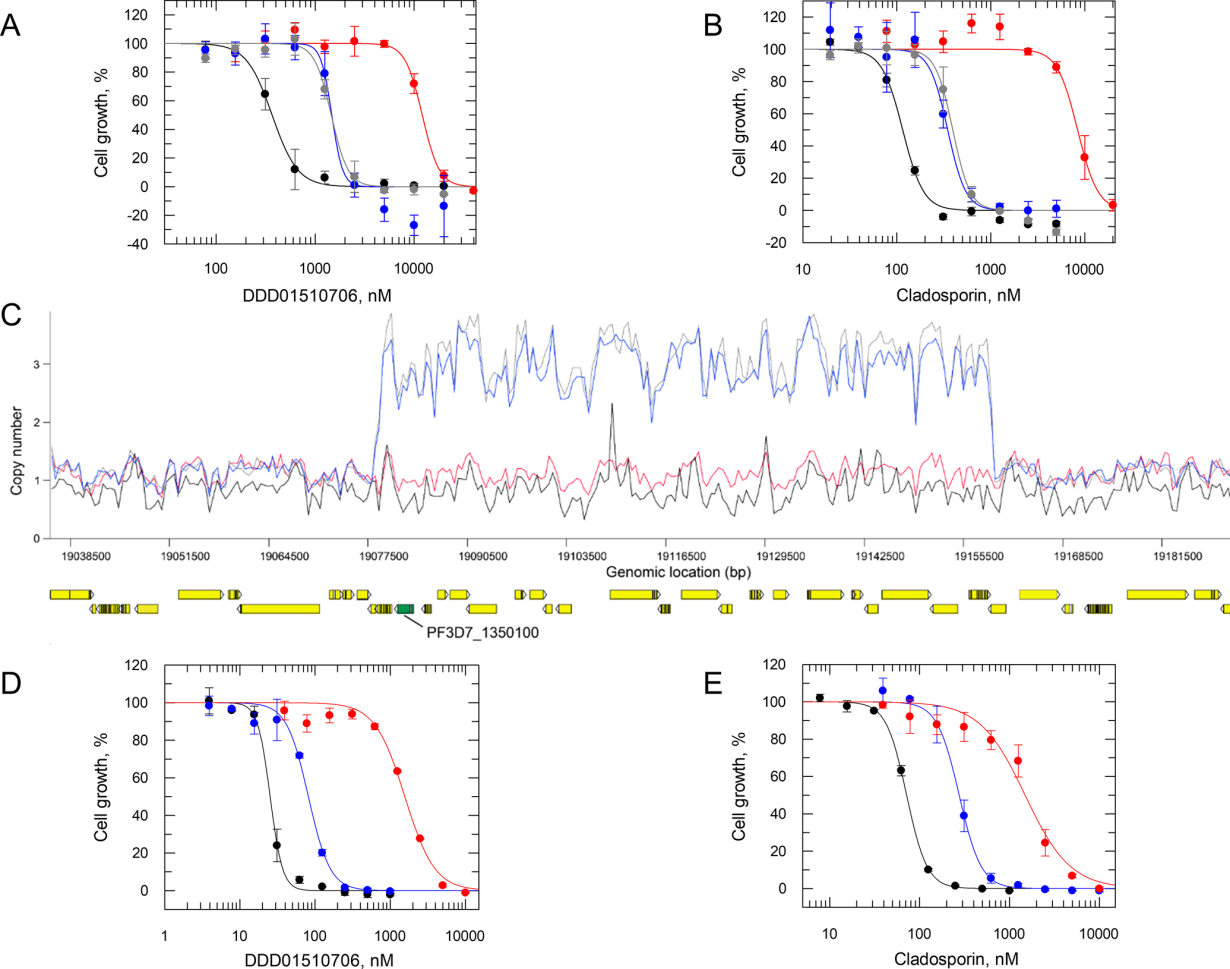
Resistance generation in vitro and analysis of DDD01510706-resistant clones. (A) EC_50_ values for DDD01510706 were determined for WT (black) and cloned resistant cell lines Res 1–3 (red, blue, and gray, respectively). An EC_50_ value of 368 ± 11 nM was determined for DDD01510706 against WT (Dd2) parasites. EC_50_ values for resistant clones Res 1–3 were 12,062 ± 592, 1472 ± 311, and 1454 ± 81 nM, respectively. (B) EC_50_ values for cladosporin were determined for WT (black) and cloned resistant cell lines Res 1–3 (red, blue, and gray, respectively). An EC_50_ value of 115 ± 8 nM was determined for cladosporin against WT (Dd2) parasites. EC_50_ values for resistant clones Res 1–3 were 8357 ± 824, 347 ± 23, and 393 ± 25 nM, respectively. (C) Copy number variations in resistant clones relative to WT. Amplification of fragments of chromosome 13 is evident in two resistant clones. Resistant clones are indicated as follows: Res 1 (red), Res 2 (blue), and Res 3 (gray); WT clone is shown in black. *PfKRS* (PF3D7_1350100) is shown in green. EC_50_ curves for DDD01510706 (D) and cladosporin (E) against WT (black), *Pf*KRS^WT^ (blue), and *Pf*KRS^S344L^ (red) overexpressing parasites. EC_50_ values of 253 ± 0.7, 819 ± 42, and 15,612 ± 1133 nM were determined for DDD01510706 against WT (NF54), *Pf*KRS^WT^ (blue), and *Pf*KRS^S344L^ (red) overexpressing parasites, respectively. EC_50_ values of 70 ± 0.09, 276 ± 7, and 14,916 ± 1621 nM were determined for cladosporin against WT, *Pf*KRS^WT^ (blue), and *Pf*KRS^S344L^ (red) overexpressing parasites, respectively. All EC_50_ curves and values are from one biological replicate, composed of two technical replicates. Collated data sets reporting the weighted mean ± SD of multiple biological replicates are summarized in Table 1.

Cell lines that are resistant to compounds in development can be of great value in guiding subsequent drug discovery.^14^ These refractory parasites can be used to rapidly profile analogues within a series to ensure that they remain on target and to prioritize analogues with a reduced resistance liability. With this in mind, we conducted in vitro evolution experiments to select for parasites resistant to the tool compound DDD01510706 (Figure 1). Drug-sensitive parasites were continuously exposed to a compound at a concentration equivalent to 3× the established EC_50_ value (600 nM) over 20 days and then cloned by limiting dilution. The resulting clones were confirmed to be between 4- and 37-fold less sensitive to DDD01510706 than the wild-type parental line (Table 1, Figure 2A). In addition, these clones were cross-resistant to the established KRS inhibitor cladosporin, with resistance ranging between 4- and 114-fold relative to wild type (Table 1, Figure 2B).

**Table 1:** Collated EC_50_ Data for Wild-Type, Resistant, and Transgenic Cell Lines

cell line	EC_50_ values,^a^ μM			
	DDD01510706	cladosporin	DDD02354914	linker 1
Dd2	0.3 ± 0.01	0.07 ± 0.003	0.4 ± 0.005	1.7 ± 0.1
Res 1	11 ± 0.5 (37)	8 ± 0.8 (114)	10 ± 0.8 (25)	25 ± 2 (15)
Res 2	1.1 ± 0.06 (4)	0.3 ± 0.03 (4)		
Res 3	1.3 ± 0.06 (4.3)	0.3 ± 0.02 (4)		
NF54-AttB	0.2 ± 0.004	0.07 ± 0.001		
KRS-OE (clone)	0.8 ± 0.006 (4)	0.3 ± 0.01 (4)		
KRS^S344L^-OE (clone)	13 ± 0.8 (65)	12 ± 1 (171)		

a All EC_50_ values represent the weighted means for at least three biological replicates (*n* ≥ 3), with each biological replicate composed of two technical replicates. Fold change in potency versus the appropriate wild-type cell line is indicated in parentheses.

Genomic DNA recovered from the three resistant clones was analyzed by whole-genome sequencing. Sequence reads were aligned to the 3D7 reference genome and compared to the wild-type parental clone. The two clones demonstrating more modest levels of resistance to DDD01510706 (Res 2 and 3) were found to have amplified an 80.7 bp fragment on chromosome 13 encompassing 23 genes including KRS (Figure 2C). These clones also shared a single nucleotide polymorphism (SNP) within a gene encoding a repetitive interspersed family of polypeptides (Table S1), part of a multigene family of variable antigens expressed on the erythrocyte surface.^15^ However, these notoriously variable surface antigens are unlikely to be involved in resistance mechanisms to DDD01510706.

The most resistant of the clones (Res 1) recovered from in vitro selections maintained a S344L mutation in KRS. Mutation of this residue is noteworthy since the published structure of cladosporin bound to *Pf*KRS revealed that the side chain of S344 forms a key part of the pocket where the cladosporin tetrahydropyran substituent binds (Figure 1).^16^ In addition, S344 alongside V328 represent the only nonconserved residues between the active sites of *P. falciparum* and human KRS enzymes.^13^

To confirm the direct roles of the S344L mutation and KRS overexpression in resistance to DDD01510706, these genomic changes were reconstituted in wild-type, drug-sensitive parasites. Cell lines overexpressing either KRS or KRS^S344L^ were generated, with elevated levels of the wild-type and mutated protein in these transgenic parasites confirmed by both quantitative RT-PCR and label-free proteomics quantification (Figure S2). In line with in vitro selected cell lines, clones overexpressing the native enzyme demonstrated modest levels of resistance (∼4-fold) to both cladosporin and DDD01510706. In contrast, clones overexpressing KRS^S344L^ showed marked resistance to both compounds (Table 1, Figure 2D).

### Chemical Pulldown

Chemical pulldown is a powerful affinity-based method to identify the protein targets of bioactive compounds. In this approach, derivatives of the compound of interest are immobilized onto a solid support in order to enrich ligand-binding proteins from a cell-free lysate. To discriminate proteins that bind specifically/nonspecifically to the affinity resin, pulldowns can be carried out in the presence of a free compound in competition. Targets specifically interacting with the immobilized derivatives are then identified and quantified by mass spectrometry (MS). Chemical pulldown in this format represents an unbiased route to identify interacting proteins, identifying primary targets as well as potential off-target liabilities.

In order to develop an immobilized version of chromone DDD01510706, we first had to identify a suitable position to attach a linker while retaining antimalarial activity. Analogues of DDD01510706 bearing an aniline methane sulfonamide at the 8-position of the chromone ring system have been reported to retain anti-parasitic activity [WO/2017/221002], and structural information supported the sulfonamide as a good vector to add a linker projecting outside the active site toward a solvent-exposed region. Therefore, we selected this position as the most appropriate one to attach our linker. Attempts to synthesize an analogue where the sulfonamide methyl group was replaced with a polyethylene glycol (PEG) chain were unsuccessful. However, a subsequent round of structure activity relationship (SAR) expansion (see Supporting Information) led to the identification of a benzene sulfonamide analogue (DDD02354914, Figure 1) that demonstrated activity comparable to DDD01510706 against asexual blood-stage (ABS) parasites and in *Pf*KRS enzymatic assays (Tables 1 and S4). DDD02354914 was further developed by introducing a PEG linker via a an amide to produce linker 1 (Figure 1). Profiling of linker 1 confirmed that, like DDD02354914, this compound is active against ABS parasites and a nM inhibitor of *Pf*KRS. Importantly, DDD01510706-resistant parasites were cross-resistant to this linker analogue (Table 1), confirming that these compounds exploit the same ATP binding site in *Pf*KRS. Further chemistry on linker 1 led to a probe molecule that was immobilized onto NHS Sepharose resin (see Supporting Information).

**Figure 3:**
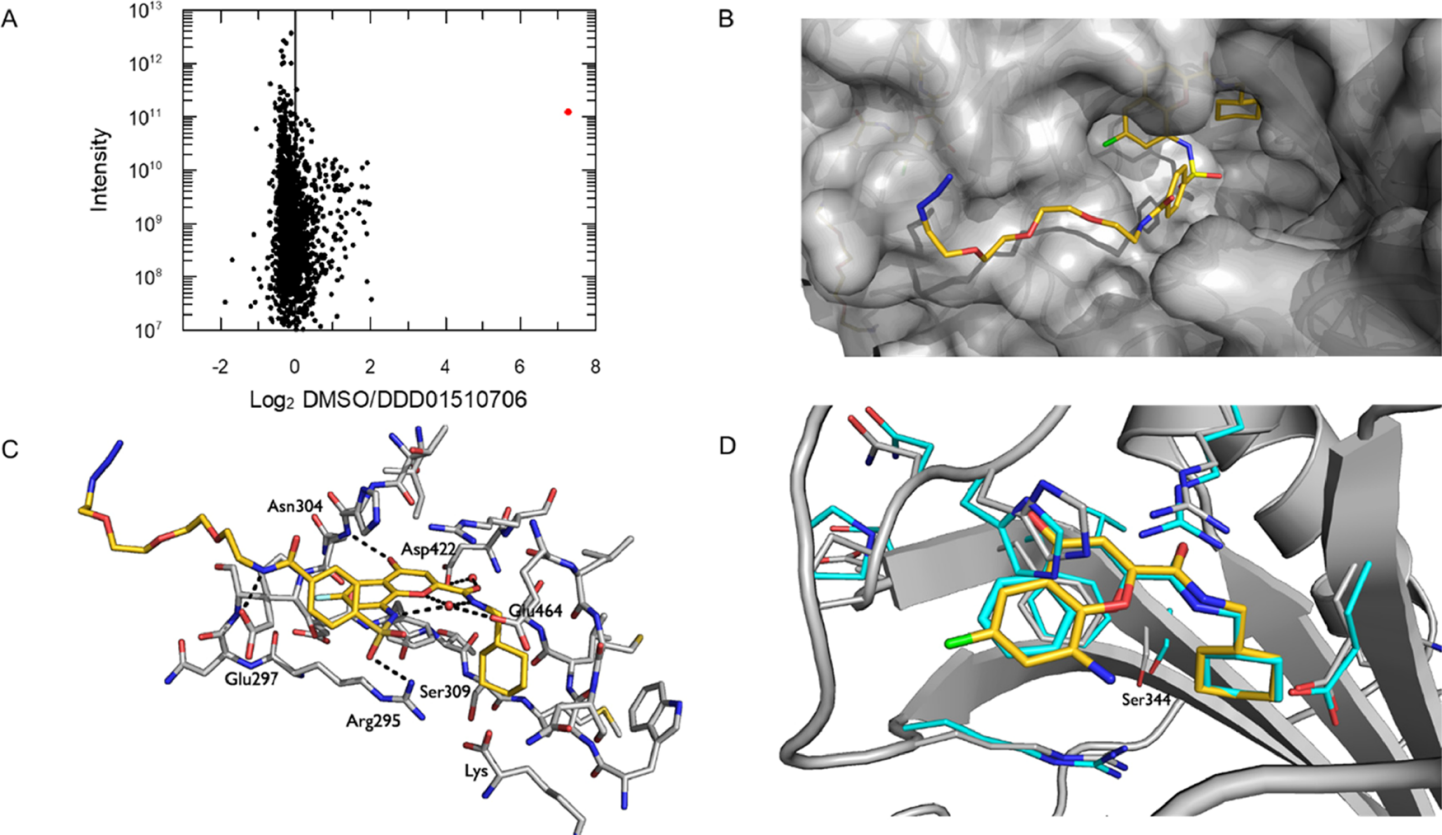
Structural analysis and chemical pulldown with a linker analogue of DDD01510706 (linker 1). (A) Differential binding of *P. falciparum* lysate-derived proteins to “low load” resin-bound linker 1 in the presence of free DDD01510706 (100 μM) or DMSO. *Pf*KRS is highlighted in red. (B) Chromone core of linker 1 binding deep into the ATP binding site of *Cp*/*Pf*KRS, while the PEG chain is exposed on the surface of the protein. The ligand is shown with carbon atoms in gold, nitrogen in blue, oxygen in red, sulfur in yellow, and fluorine in green. (C) Close-up of the interactions formed between linker 1 and *Cp*/*Pf*KRS. Potential interactions are represented with dashed lines. All residue numbers refer to the *Cp*/*Pf*KRS sequence. Please note that S309 is equivalent to S344 in *Pf*KRS. (D) Overlay of the active sites of *Cp*/*Pf*KRS-ligand and *Pf*KRS with a chromone ligand bound (PDB:6agt). The overlay is based on the bound lysine in the active site. For clarity, the benzene sulfonamide and PEG part of the ligand have been omitted. *Pf*KRS is shown with cyan carbon atoms, *Cp*/*Pf*KRS with gray carbon atoms, and the ligand with gold carbons.

Our previous experience indicates that the level of probe loaded onto beads can materially impact the outcome of the subsequent pulldown. In light of this observation, our standard practice is to carry out pulldowns using both “high” and “low” loaded beads. In this instance, bead loading was confirmed at 82 and 27% of maximum capacity (23 μmol/mL resin) for high and low loaded beads, respectively. Pulldowns with both resins in the presence and absence of competition from DDD01510706 led to significant differential enrichment of *Pf*KRS (Figures 3A and S3). As expected, this indicates that *Pf*KRS is the principal target of the chromone inhibitor series. Relatively few additional proteins were enriched in these pulldowns, particularly those using low loading level beads, demonstrating the specificity of the interaction between DDD01510706 and *Pf*KRS.

### Hybrid *Cp*/*Pf*KRS Crystal Structure Suitable for Ligand Soaking

High-resolution cocrystal structures of ligands bound to their molecular targets can facilitate drug discovery by enabling structure-based drug design. While high-resolution structures of *Pf*KRS have been reported,^13,16^ we have had difficulty in generating robust and reproducible crystal structures to support our *Pf*KRS drug discovery platform. To increase throughput and reliability and to reduce compound to structure timelines, a chimeric protein based upon *Cryptosporidium parvum* KRS was considered. *Cp*KRS is known to be structurally tractable, and protein–ligand cocrystal structures are readily generated.^13^ The structures of *Pf*KRS and *Cp*KRS in complex with cladosporin were superimposed for analysis (Figure S4); of the 40 residues defining the cladosporin binding site, 9 were not conserved between the two enzymes. Visual inspection of the superimposed structures identified just three *Cp*KRS residues that alter the shape and nature of the binding pocket relative to *Pf*KRS; *Cp*KRS^Pro272^ equivalent to *Pf*KRS^Thr307^, *Cp*KRS^Asn293^ equivalent to *Pf*KRS^Val328^, and *Cp*KRS^Ala309^ equivalent to *Pf*KRS^Ser344^. The side chains of the remaining six nonconserved residues do not extend into the ligand-binding site, and the low RMSD between the atomic positions suggests that these residues have a minimal effect upon binding site conformation. Based on this information, Pro272, Asn293, and Ala309 in *Cp*KRS were mutated to match the equivalent *Pf*KRS residues. This hybrid protein rapidly produced crystals suitable for ligand soaking with high reproducibility and high diffraction quality. *Cp*/*Pf*KRS now represents a key reagent supporting our ongoing drug discovery program, enabling high-resolution structural information to be provided in rapid time-frame facilitating structure-guided compound optimization. The use of structural information, combined with understanding of the ligand-binding interactions, can be used to drive the design of highly selective inhibitors. As we have already stated,^13^ it is likely that the *Plasmodium* and human KRS enzymes have different hydrogen bond networks, which can give rise to ligand selectivity; in this case, it is due to different configurations of the two binding sites and different degrees of stabilization of the enzymes on ligand-binding. We intend to use this high-resolution structural information to better exploit these differences for future inhibitor design.

*Cp*/*Pf*KRS crystals soaked with linker 1 yielded a high-resolution (1.9 Å) ligand-bound structure (Figure 3B–D). Initial refinement of the structure indicated that the presence of the large PEG linker did not affect the binding of the chromone core of linker 1 in the ATP pocket, with the benzene sulfonamide and PEG linker extruding from the active site toward the protein surface (Figure 3B). The chromone core and benzene sulfonamide are well defined in the electron density; however, the solvent-exposed terminal end of the PEG chain is less well defined (Figure S5). The chromone core maintains all anticipated interactions with the protein (Figure 3C), with additional interactions formed between the benzene sulfonamide and Arg295 and Glu297. Side chain positions are maintained with respect to the chromone-bound structure of *Pf*KRS (Figure 3D). Interestingly, the PEG side chain only appears to show limited interaction with the *Pf*KRS protein, indicating that most of the binding is driven through interactions between the chromone in the ATP binding site. Thus, the PEG-linked chromone represents an excellent tool for pulldown and competition studies to identify compounds that bind in the ATP site.

### Thermal Proteome Profiling

**Figure 4:**
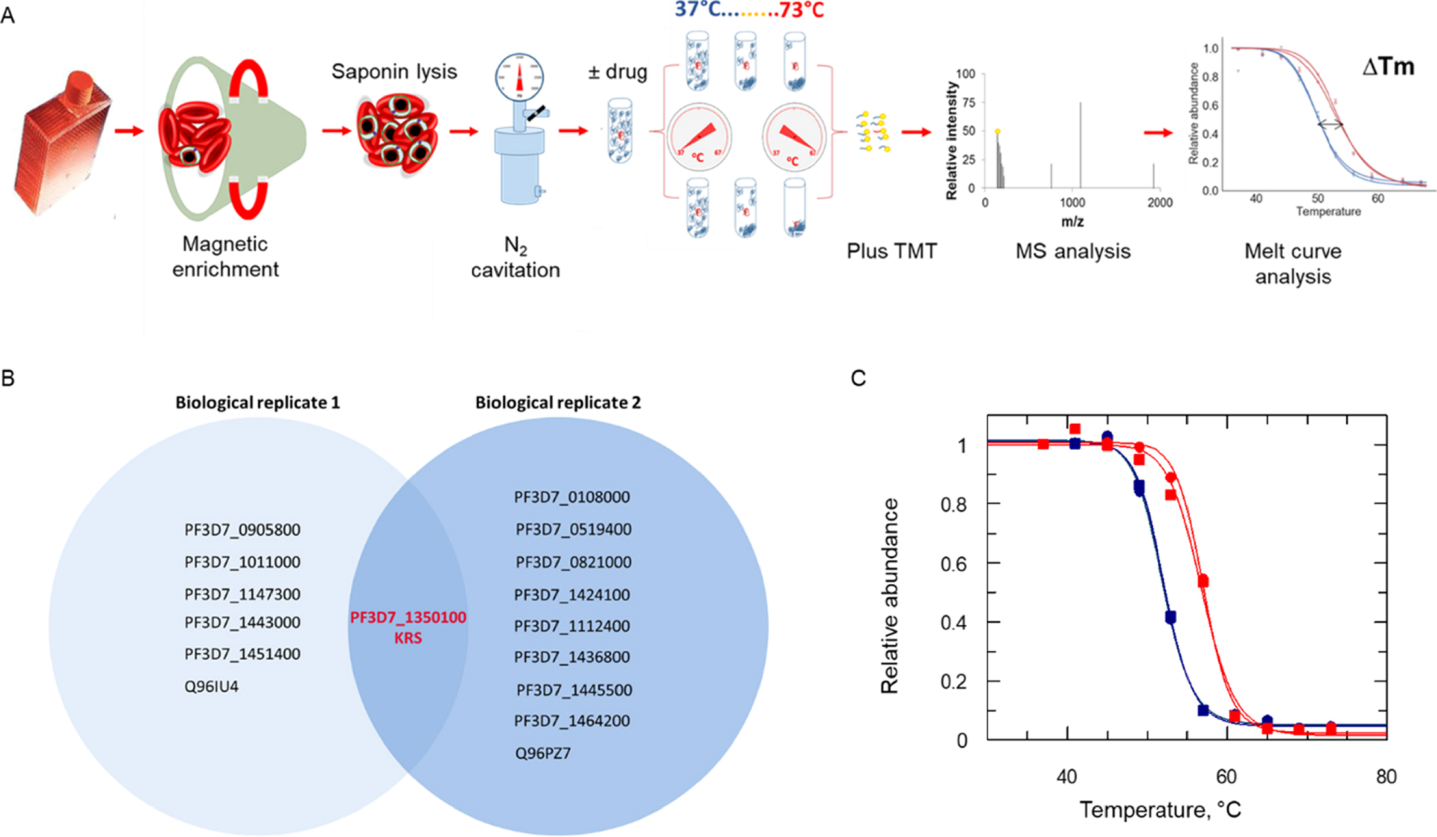
Target deconvolution utilizing TPP. (A) Schematic representing our TPP workflow. TMT: tandem mass tags. (B) Venn diagram of proteins displaying the most significant thermal shift in the presence of DDD01510706 from duplicate experiments (biological replicates). PlasmoDB gene IDs (*P. falciparum* proteins) and Uniprot identifiers (human proteins) are used to represent individual proteins. (C) Melt curves for *P. falciparum* LysRS following incubation with 3.4 μM DDD01510706 (red) or vehicle (0.1% DMSO, blue) in the two experiments (biological replicates). Data from two technical replicates (circles and squares) are shown, and the mean shift in melting temperature (Δ*T*_m_) for KRS was 5.6 °C. Data from an independent duplicate experiment are presented in Figure S6 and Table S8.

Thermal proteome profiling (TPP) can be used as an alternative, unbiased approach to demonstrate compound–target engagement. It is based on the principle that binding of a drug to its protein target can significantly alter the thermal stability of that protein.^17^ Here, we adapted our standard TPP strategy^18^ to investigate interaction of DDD01510706 with proteins within a *P. falciparum* cell lysate (Figure 4A). To reduce the levels of contaminating red blood cell protein identified in order to prioritize the depth of parasite proteome visible in our subsequent analysis, harvested cell material was subjected to magnetic enrichment to isolate erythrocytes infected with late trophozoite and schizonts, while uninfected red blood cells were discarded. Infected red blood cells were lysed by exposure to saponin prior to lysis of parasites by N_2_ cavitation. ABS lysates were exposed to either DDD01510706 at 10× its established EC_50_ value or DMSO for 30 min. Aliquots of the resulting drug-treated and control parasite lysates were incubated at designated temperatures (37 to 73 °C), and then insoluble (denatured) proteins were removed. The resulting soluble protein samples were reduced, alkylated, and digested with trypsin and LysC prior to derivatization with tandem mass tags (TMT). Pooled peptides were fractionated by high-pressure liquid chromatography and analyzed by LC−MS/MS prior to identification and quantitation. The melting points of identified proteins were then established using the TPP software package. Full melt curves were established for 2644 proteins, representing 49.1% coverage of the theoretical *P. falciparum* proteome. This coverage compares well with the 46.2 and 36.4% proteome coverage reported for previous TPP and cellular thermal shift assay coupled with MS studies with *P. falciparum*.^19,20^

TPP studies with DDD01510706 were carried out in biological replicate with six *P. falciparum* proteins identified as putative targets in replicate 1 and nine identified in replicate 2 (Figure 4B). However, the only target candidate that demonstrated increased thermal stability in the presence of DDD01510706 across both data sets was *Pf*KRS. Individual melting curves revealed that the thermal stability of *Pf*KRS increased by 5.6 °C (mean Δ*T*_m_) in experiment 1 (Figure 4C) and by 4.8 °C in experiment 2 (Figure S6; see also Tables S7 and S8). It should be noted that while numerous tRNA synthetases were identified in the MS analysis of our *P. falciparum* lysates, none of these enzymes were identified as possible targets of DDD01510706, indicating the selectivity of this inhibitor. Collectively, these data again confirm *Pf*KRS as the primary target of DDD01510706 and illustrate the utility of TPP in confirming on-target engagement of compounds within a biologically relevant milieu. This approach can be considered complementary to chemical pulldowns since it does not require compound modification and can be particularly useful in the absence of structural information or SAR.

### Isothermal TPP

**Figure 5:**
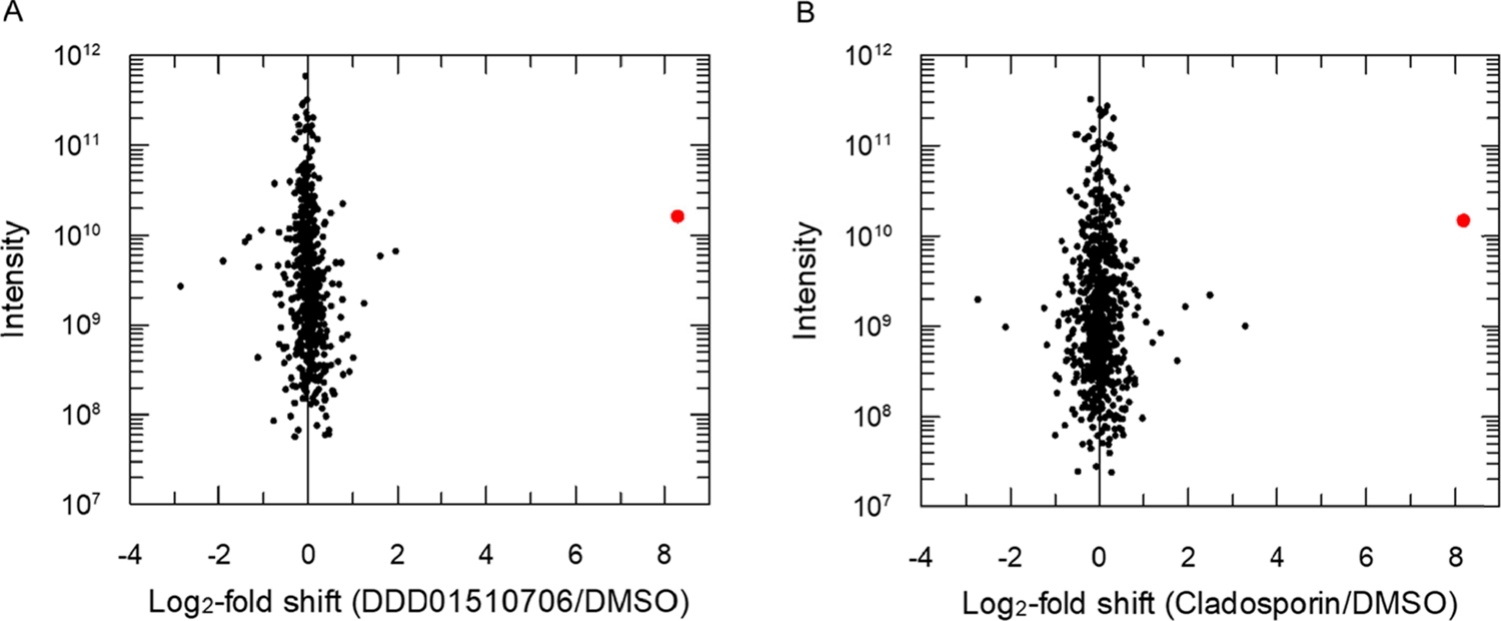
Isothermal TPP for proteome scale drug-target identification. Plots show protein abundance log_2_ fold change between compound-treated and untreated lysates subjected to thermal shock at 57 °C. Lysates exposed to DDD01510706 (A) and cladosporin (B). Data are sorted by protein total intensity on the *y*-axis. Proteins identified with <2 unique peptides are shown as squares and proteins >2 unique peptides as circles. *Pf*KRS is indicated in red.

Although powerful and effective, TPP in the format described above can be time-consuming, expensive, and unsuitable for high throughput. To ensure analogues emerging from our *Pf*KRS drug discovery program remain on-target and to efficiently exclude problematic compounds with undesirable off-target profiles, we developed a simplified isothermal TPP methodology (iTPP). In this rationalized assay, the relative abundance of proteins in *P. falciparum* cell lysates is monitored at a single temperature in the presence and absence of test compounds rather than across a broad temperature range. Increased abundance of a specific protein in the presence of a compound is indicative of thermo-stabilization of the target as a result of a direct interaction with the ligand. Based on the results of our full TPP analysis, we chose to profile the thermal stability of proteins in the presence/absence of DDD01510706 at 57 °C. At this temperature, the abundance of *Pf*KRS, determined by label free quantitation (LFQ), was elevated in the presence of DDD01510706, while the levels of other proteins within the lysate were not significantly altered (Figure 5A). The relative abundance of *Pf*KRS was also significantly increased in lysates exposed to cladosporin (Figure 5B). The simplified experimental design of iTPP lends itself to the high throughput assessment of analogues in development. Chemical labeling with isobaric TMT also enables the analysis of samples to be multiplexed. This approach can be readily adapted to identify targets in live cells as well as cell lysates. Thus, iTPP represents a streamlined approach for proteome-wide identification of ligand binding.

## Conclusions

To date, the vast majority of anti-malarial discovery programs have focused on developing hits identified through phenotypic screening. This is in part due to the success of phenotypic approaches but also partly enforced by the lack of robustly validated drug targets in *Plasmodium.* However, the emergence of improved genetic tools for the study of this apicomplexan parasite has enabled many more viable drug targets to be identified and robustly validated.^6,21,22^ This, coupled with the fact that the massive screening efforts of organizations such as Medicines for Malaria Venture has explored and now exhausted much of the available chemical space, has led to a renewed focus on target-based drug discovery. To support this decided change of emphasis, the development of additional tools and methodologies will be required. The diverse toolkit of approaches that we have developed and outlined in this paper has proved invaluable in supporting our *Pf*KRS focused studies. We believe that this strategy can be readily replicated and applied to other target-based anti-malarial drug discovery programs.

## Methods

### Cell Lines and Culture Conditions

*Plasmodium falciparum* ABS parasites (strains 3D7, Dd2, and NF54-AttB) were cultured as previously described.^23^ Briefly, parasites were maintained in A^+^ human erythrocytes sourced from Scottish Blood Transfusion Service with a hematocrit of 5%. Infected red blood cells were cultured in Complete Malaria Media (CMM; RPMI 1640 media [Gibco] supplemented with 25 mM HEPES, 2 mM l-glutamine, 0.5% albumax II [Gibco], 12 mM sodium bicarbonate, 0.2 mM hypoxanthine, and 20 mg/L gentamicin, pH 7.3) at 37 °C in a humidified atmosphere of 1% O_2_ and 3% CO_2_ in a balance of N_2_. Parasitaemia was maintained between 1 and 5% with daily media changes and with the addition of fresh red blood cells every 48–72 h. When required, cultures were synchronized by two rounds of d-sorbitol treatment, as previously described.^24^

### Drug Sensitivity Assays

The relative potency of test compounds was determined using a SYBRGreen-based assay.^13^ Cultures (2.5% hematocrit and 0.3% parasitaemia) were exposed to test compounds over a range of concentrations (doubling dilutions) in 96-well plates and incubated for 72 h. SYBRGreen reagent in lysis buffer [20 mM Tris–HCl, 5 mM EDTA, 0.16% w/v saponin, 1.6% (v/v) Triton X-100, pH 7.9] was then added to one times concentration, and plates were incubated at room temperature and protected from light for 4–24 h. Fluorescence (excitation 485 nm and emission 528 nm) was measured on a Tecan Infinite Pro 200 microplate reader. Data were fitted to the two-parameter equation from GraFit version 7.0 (Erithacus Software), and EC_50_ values were calculated. Treatment with the standard inhibitor mefloquine (10 μM) was used to define 0% parasite growth.

### Resistance Generation

A total of 10^9^*P. falciparum* Dd2 parasites were exposed to DDD01510706 (600 nM). Every 2–3 days the media (supplemented with fresh compound) were refreshed and the culture inspected for parasite growth regularly. After 20 days, parasites were visible and subsequently cloned by limiting dilution. For DNA isolation, the parasite cultures were centrifuged (10 min, 1800*g*, RT) and the resulting pellet was resuspended in 0.15% (v/v) saponin in PBS (5× pellet volumes) and incubated for 5 min at RT. After washing the parasite pellet with incomplete malaria media (IMM), DNA was isolated using standard alkaline lysis.

### Parasite Cloning

Parasites were added to 96-well plates at a density of 0.5 parasites/well and a 2% hematocrit. The plates were incubated for 17–19 days in standard conditions, and the media was changed every 7 days with the addition of 0.4% fresh red blood cells. To identify positive wells, 40 μL from each well of the cloning plate was transferred to a fresh plate and SYBRGreen reagent in lysis buffer (see above) was added. After incubation for >1 h, fluorescence was measured (excitation 485 nm and emission 528 nm). Wells confirmed to contain live parasites were subcultured into fresh culture media at a 5% hematocrit.

### Whole-Genome Sequencing

DNA was sequenced on an HiSeq X Ten machine by the Beijing Genomics Institute. Sequencing reads were mapped to the 3D7 reference genome (version 48, https://plasmodb.org) using bowtie2 (version 2.3.5),^25^ with the option “-very-sensitive-local.” SAM alignment files were converted to binary files, sorted, and indexed using SAMtools (version 1.9^26^). Sequence variants were called using SAMtools bcftools mpileup (options -d8000 -C50) and bcftools call (options -cv -f GQ).^27^ For higher throughput, variant calling was run on multiple cores using GNU parallel. The obtained sequence variants were annotated with snpEff (version 5.0)^28^ using the 3D7 reference sequence and GFF annotation file (version 48, https://plasmodb.org). The resulting VCF file was processed with python scripts, whereby variants that were (1) nonsynonymous (predicted to have a “MODERATE” or “HIGH” impact by snpEff); (2) absent in the Dd2 parent cell line (called as 0/0, with a genotype quality >20); and (3) present in at least one of the resistant clones were extracted and considered as candidate variants. These candidate variants were visually inspected in the Integrative Genomics Viewer (version 2.7.0^29^) to exclude false positives.

To identify larger copy number variants, the RPKM values of all coding sequences were analyzed with the Artemis genome browser (version 16^30^). RPKM values of the resistant clones were normalized with the RPKM values of the Dd2 parent line, plotted with ggplot2 in R and inspected visually for copy number alterations. All whole-genome sequencing data produced in these studies have been deposited with the European Nucleotide Archive under accession number: **PRJEB47416**.

### Generation of Overexpression Constructs

For overexpression of *P. falciparum* KRS (LysRS; PF3D7_1350100), the BxbI viral integrase system was used to stably integrate the LysRS coding sequence (CDS) under the control of the calmodulin promoter into the nonessential *cg6*-locus.^31^ The *Pf*KRSRS and *Pf*KRS^S344L^ CDS were custom-synthesized (GeneArt) and cloned into the pDC2-cam-mRFP-2A-GFP-bsd-attP vector^32^ via AvrII/XhoI sites. The resulting vectors (pDC2-cam-KRS-bsd-AttP and pDC2-cam-KRS^S344L^-bsd-AttP) were cotransfected with the pInt plasmid^31^ into the NF54-AttB cell line. All primers used in this aspect of the study are summarized in Table S1.

### Transfection

All transfections were performed using a Lonza Nucleofector 4D. After sodium acetate ethanol precipitation, plasmid DNA (50 μg/transfection) was resuspended in 100 μL of Nucleofector buffer P3 (Lonza) supplemented with 12.5 mM ATP on the day of transfection. Ring-stage parasites (parasitaemia 5–10%, 2.5% hematocrit) were used for transfections (100 μL of infected red blood cells/transfection). Cells were washed once in incomplete cytomix (120 mM KCl, 0.15 mM CaCl_2_, 10 mM K_2_HPO_4_/KH_2_PO_4_, 25 mM HEPES, 2 mM EGTA, 5 mM MgCl_2_, pH 7.6), and the resulting pellet was resuspended in the DNA/P3/ATP mix and then divided between two nucleofection cuvettes. Following electroporation (program CM150), cells were incubated on ice for 1 min before being transferred to a 15 mL Falcon tube containing 10 mL of complete media/5% red blood cells prewarmed to 37 °C. Following 4 h recovery at 37 °C, cells were pelleted (1000*g*, 5 min, RT, low brake), resuspended in fresh prewarmed CMM, and incubated overnight. Antibiotics selection was initiated 24 h after transfection, with antibiotics added to cultures at the following concentrations: 250 μg/mL G418 and 2 μg/mL blasticidin S. Media (supplemented with appropriate antibiotic selection) were changed daily for 6 days and then every second day until viable parasites were observed. Fresh RBCs (50 μL/10 mL culture) were added at 6 and 11 days post-transfection. At 2, 3, and 4 weeks post-transfection, one-third of the culture was discarded and replaced with fresh media/RBCs to mitigate RBC lysis. The resultant transfectants were cloned by limiting dilution.

### Quantitative-RT PCR

Following two rounds of d-sorbitol treatment, RNA was isolated from synchronized trophozoite-stage parasites (24 h post-treatment) using a RNeasy mini kit (Qiagen), according to manufacturers’ instructions. Quantitative-RT PCR was performed using the Luna One Step RT-qPCR kit (NEB) on an Agilent MX3005P system. mRNA levels were normalized to the reference gene, β actin (PF3D7_1246200), and expression levels compared using the ΔΔC_t_ method. Primers used are detailed in Table S1.

### Label-Free Quantification

Lysates used for analysis via label-free quantitation were prepared as described for TPP. Relative protein abundance in WT versus overexpressing cell lines was established as previously described^33^ and normalized to β actin. In this instance, proteins were identified by searching the *P. falciparum* 3D7 proteome (plasmodb.org, version 49).

### *P. falciparum* Lysate Preparation—Thermal Proteome Profiling

For cultures that were ultimately used to prepare TPP lysates, synchronized parasites were grown in HYPERFlasks (Corning) and the hematocrit maintained at 1.5–2% with daily or twice daily media changes. Once cultures reached between 8 and 15% parasitaemia, they were harvested by centrifugation (1800*g*, RT, 15 min, low brake) and the resulting pellets were washed once and then resuspended in 5× pellet volumes of IMM (incomplete malaria media; as CMM but without albumax II). Late trophozoite/schizont-infected erythrocytes were isolated via MACS separation using a SuperMACS II magnet in conjunction with a D column (Miltenyi Biotec). First, the D column was equilibrated with 10× column volumes of phosphate-buffered saline followed by 3× column volumes of IMM. Next, infected erythrocytes were passed through the column 3× via a 20-G needle at a flow rate of 1 drop per second. The column was rinsed with IMM until the flow-through ran clear and then removed from the magnet. Trophozoite/schizont-infected erythrocytes were eluted from the column by passing one column volume of IMM through the column 5–6× using a 20 mL syringe. Following centrifugation (1800*g*, 5 min, low break), parasitized erythrocytes were lysed by incubation in 0.1% (w/v) saponin on ice for 10 min with gentle mixing. Free (or extracellular) parasites were harvested by centrifugation (2800*g*, 10 min at 4 °C) and washed 3× in wash buffer (WB; 100 mM potassium acetate, 2.5 mM magnesium acetate, 45 mM HEPES [pH 7.4], 250 mM sucrose, 2 mM dithiothreitol, 15 μM leupeptin) to remove lysed red blood cell material. The pellet was resuspended in one volume of WB supplemented with a protease inhibitor (Roche cOmplete EDTA-free protease inhibitor; 1 tablet/20 mL), and the parasites were lysed by nitrogen cavitation (Parr) (4 °C, 1500 psi, 60 min). The resulting lysate was centrifuged (100,000*g*, 20 min, 4 °C), the supernatant was harvested, and the protein concentration of the lysate was determined using the Bio-Rad Protein Assay.

### TPP Assays

TPP assays were performed as previously described.^18^ However, in this instance, lysates were exposed to the following temperature range: 37, 41, 45, 49, 53, 57, 61, 65, 69, and 73 °C.

### Sample Processing, Fractionation, Protein Identification, and Quantitation

All aspects of sample processing, TMT labeling, fractionation by HPLC and LC–MS/MS, and protein identification and quantitation were described previously.^18^ Proteins were identified by searching the MS and MS/MS data for the peptides against *P. falciparum* strain 3D7 (Plasmo DB version 45, plasmodb.org) using the software MaxQuant (http://maxquant.org/, version 1.6.1.0). All proteomics data sets have been deposited to the ProteomeXchange Consortium via the PRIDE^34^ partner repository with the identifier **PXD025182**.

### TPP Data Analysis

TPP experiments were analyzed using the TPP Package available in Bioconductor, as previously described.^18,35,36^ Briefly, raw protein abundance, calculated from the normalized reporter ion intensities of all quantified proteins, were normalized to the protein abundance at the lowest temperature for each condition and replica. Melting curves were calculated using a sigmoidal fitting algorithm in the TPP Package of the R program. This fitting was used to determine the melting point (*T*_m_), which is defined as the temperature in which half of the protein was denatured. The melting point differences (Δ*T*_m_) were calculated by subtracting the *T*_m_ values of treated and untreated samples. The sigmoidal melting curves were filtered according to the following criteria: melting curves must reach a relative abundance plateau < 0.3 and the coefficient of determination (*R*^2^) must be >0.8. The statistical significance was calculated using a *z*-test, and only proteins with a *p*-value <0.01 were considered hits. Hits found in two biological replicas were considered putative targets.

### Isothermal TPP

Isothermal TPP assays were performed as described for standard TPP assays, but in this instance, lysates were incubated at two temperatures: 37 and 57 °C. Quantification was achieved using LFQ. The first temperature (37 °C) acts as a control temperature at which the levels of KRS in samples are not altered by the presence of the test compound. The second temperature (57 °C) was selected on the basis that KRS is significantly enriched in samples incubated in the presence DDD01510706 in comparison to samples incubated in the absence of compound.

### *P. falciparum* Lysate Preparation—Pulldown

An asynchronous culture was used for pulldown analysis. Cells were harvested by centrifugation (1800*g*, 15 min, RT, low brake) and washed once with IMM, and then red blood cells were lysed by incubation in 0.1% (w/v) saponin in PBS (10 min on ice with gentle shaking). Following 3× washes in ice-cold WB [100 mM potassium acetate, 2.5 mM magnesium acetate, 45 mM HEPES (pH 7.4), 250 mM sucrose, 2 mM dithiothreitol, 15 mM leupeptin], the pellet was resuspended in one volume of lysis buffer [WB supplemented with protease inhibitor (Roche cOmplete EDTA-free protease inhibitor; 1 tablet/20 mL) and 0.8% (w/v) Octyl β-d-glucopyranoside]. The parasites were then lysed by nitrogen cavitation (Parr) on ice at a pressure of 1500 psi for 1 h.

### Preparation of Drug Beads for Chemical Pulldown

Two loading levels of drug bead (termed “high” and “low”) were prepared in parallel. Please note that the Cytiva NHS-activated Sepharose 4 Fast Flow resin used in this preparation has a quoted concentration of 16–23 μmol NHS groups/mL settled resin. All steps were carried out at RT.

Stock solutions for high loading (A) and low loading (B) as follows: A—138 μL of 50 mM **6** in DMSO, 138 μL of 100 mM DIPEA in DMSO and DMSO (24 μL) (final volume 300 μL); B—27.6 μL of 50 mM **6** in DMSO, 27.6 μL of 100 mM DIPEA in DMSO and DMSO (244.8 μL) (final volume 300 μL). A suspension of Cytiva NHS-activated Sepharose 4 Fast Flow resin (400 μL, approx. 1.1 slurry in isopropanol) was centrifuged at 15,000*g* for 30 s, and the supernatant was decanted. DMSO (1 mL) was added to the pelleted resin, gently mixed, and centrifuged at 15,000*g* for 30 s, and the supernatant was removed. Washing steps were repeated three times. Stock solution (A or B, 200 μL) was then added to the washed Sepharose resins and gently mixed for 24 h at RT. Reactions were then centrifuged at 15,000*g* for 30 s, supernatants were removed, and the resin was washed three times with DMSO (1 mL) and once with a solution of ethanolamine (200 μL, 200 mM in DMSO). The pelleted drug beads were then treated with an ethanolamine solution (200 μL, 200 mM in DMSO) with mixing for 24 h at RT. This ethanolamine treatment “caps” any unreacted NHS esters. The resin was then centrifuged (15,000*g*, 30 s) and washed with DMSO (3 × 1 mL) to complete the drug bead synthesis. “Blank beads” were prepared by treating washed resin with ethanolamine solution as described above.

The loading level of drug bead batches was estimated by monitoring the disappearance of **6** from the reaction solution using LCMS. Assuming the Sepharose resin contains 23 μmol NHS groups/mL, the “high loading” and “low loading” drug beads were estimated to have loading levels of 82 and 27%, respectively. Resins were stored at 4 °C as 1:1 suspensions in iPrOH (approximately 200 μL iPrOH/200 μL of resin). The pulldown experiments were carried out using these iPrOH suspensions.

### Chemical Pulldown

Parasite lysates were centrifuged (20,000*g*, 4 °C, 15 min). Beads (blank and with linker attached) were washed 3× with water and then twice with lysis buffer. The lysate (∼2 mg total protein) was first incubated with blank beads for 30 min at 4 °C with rotating agitation to remove proteins binding nonspecifically to the beads. The lysate was divided into two aliquots and then incubated with either 1% DMSO or 100 μM DDD01510706 (competitor) for 30 min at 4 °C with agitation. Finally, lysates were incubated with compound-bound beads (“high” or “low” loading) for 1 h at 4 °C with agitation. The beads were then washed 3× with WB (0.8% (w/v) octyl β-d-glucopyranoside, 50 mM Tris pH 8.0, 5 mM EDTA, 1 mg/mL BSA) and 2× Tris-buffered saline (TBS; 50 mM Tris-Cl pH 7.5, 150 mM NaCl). Samples were run 1.5 cm into a Bis-Tris 10% (w/v) acrylamide gel and stained with Coomassie quick reagent for 30 min. The entire gel bands were removed and subjected to in-gel reduction with 10 mM dithiothreitol, alkylation with 50 mM iodoacetamide, and digestion with 12.5 μg/mL trypsin (Pierce) for >16 h at 37 °C. Recovered tryptic peptides were then vacuum-dried prior to analysis.

### Pulldown Proteomics Analysis

TMT-labeling and fractionation were performed as described above. Analysis of peptides was performed on an Orbitrap Eclipse (Thermo Scientific) mass spectrometer coupled to a Dionex Ultimate 3000 RS (Thermo Scientific). Online HPLC was performed as previously described.^18^ Orbitrap Eclipse was used in data-dependent mode. A scan cycle comprised MS1 scan [*m*/*z* range from 380–1500, with an automatic maximum ion injection time, a resolution of 120,000, and a standard automatic gain control (AGC) target value] followed by sequential dependent MS2 scans (with an isolation window set to 0.7 Da, maximum ion injection time at 50 ms, and standard AGC target) and MS3 scans (with a resolution of 50,000, an isolation window set to 0.7 Da, maximum injection time at 120 ms, and 400% AGC target). The real-time search feature was active during the analysis.

Analysis of the resulting MS data was performed using the software MaxQuant (http://maxquant.org/, version 2.0.3.0). Modifications, digestions, and database search settings were as previously described. Reporter ion MS3 mode was selected using the TMT-10plex labels on N-terminus and lysine. FTMS MS/MS mass tolerance was set to 10 ppm, and ITMS MS/MS mass tolerance was 0.5 Da.

All MS data associated with chemical pulldowns have been deposited to the ProteomeXchange Consortium via the PRIDE^34^ partner repository with the data set identifier **PXD033740**. Data were analyzed with Perseus (version 1.6.15^37^). The reporter intensity of each protein was extracted and used to calculate the log_2_-transformed fold changes of DMSO versus competitor-treated samples.

### *Pf*KRS and *Hs*KRS Assays

Purified recombinant *Pf*KRS and *Hs*KRS used in enzymatic assays were produced as previously described.^13^ The activities of *Pf*KRS and *Hs*KRS were determined by monitoring levels of pyrophosphate released during the first step of the KRS enzymatic reactions. The pyrophosphate formed was converted to two inorganic phosphate molecules using a pyrophosphatase enzyme and levels of the resulting phosphate molecules measured using the BIOMOL Green reagent (Enzo Life Sciences). All screening assays were performed in 384-well, clear, flat-bottom plates (Greiner) at room temperature (∼23 °C) in 50 μL reaction volumes.

*Pf*KRS assay wells contained *Pf*KRS assay buffer (100 mM Hepes; pH 7.4, 100 mM NaCl, 20 mM MgCl_2_, 0.01% (v/v) Igepal and 1 mM DTT) plus 20 nM *Pf*KRS, 200 μM ATP, 400 μM l-lysine, and 0.5 U/mL pyrophosphatase. *Hs*KRS reactions contained *Hs*KRS assay buffer (30 mM Tris; pH 8, 140 mM NaCl, 40 mM MgCl_2_, 30 mM KCl, 0.01% (v/v) Brij and 1 mM DTT) plus 400 nM *Hs*KRS, 3.5 μM ATP, 6 μM l-lysine, and 0.5 U/mL pyrophosphatase. Assays were performed by adding 25 μL of assay buffer with enzyme to all assay wells, before the reaction was initiated with the addition of a 25 μL substrate mixture containing l-lysine, ATP, and pyrophosphatase (a substrate mix with l-lysine omitted was added to “no lysine” control wells on each assay plate). Following a 6 h (*Pf*KRS) or 3 h (*Hs*KRS) reaction at RT, the assay was stopped with the addition of 50 μL BIOMOL Green. The BIOMOL Green signal was allowed to develop for 30 min before the absorbance of each well was read at 650 nm using an EnVision multilabel plate reader (PerkinElmer Life Sciences) or a PheraStar plate reader (BMG). All liquid dispensing steps were carried out using a Thermo Scientific WellMate dispenser (Matrix).

To generate pIC_50_ data for hit compounds in the *Pf*KRS or *Hs*KRS assays, 10-point inhibitor dose–response curves were prepared in 384-well assay plates using an ECHO 550 acoustic dispenser (Labcyte). Following preparation of the inhibitor curves, assays were carried out as described above. ActivityBase from IDBS (version 8.0.5.4) was used for data processing and analysis, with percentage inhibition values determined relative to 100% inhibition (“no-lysine” control) and 0% inhibition control wells on each plate. All IC_50_ curve fitting was undertaken using ActivityBase XE (version 7.7.1) from IDBS. A four-parameter logistic dose–response curve was utilized (XLfit model 203) with prefit used for all four parameters.

### Recombinant Expression and Purification of *Cp*/*Pf*KRS

DNA encoding residues 45–559 of *Cp*KRS with three mutations (P272T, N293V, and A309S) and codon-optimized for expression in *E. coli* was synthesized (GenScript) and introduced into a modified version of the pET15b vector, with an N-terminal 6 × His tag and a Tobacco Etch Virus cleavage site. The resulting plasmid was transformed into *E. coli* BL21(DE3) (Stratagene). Starter cultures were grown overnight at 37 °C in LB media with carbenicillin and used to inoculate 12 L of LB Autoinduction media (10 mL starter per liter). Cultures were grown for 48 h at 20 °C. Cells were harvested by centrifugation (3500*g*, 20 min, 4 °C), and the resulting pellets were stored at −20 °C.

Cell pellets were thawed on ice and then resuspended in lysis buffer (25 mM HEPES, 150 mM NaCl, 5% glycerol, 0.5 mM TCEP, 20 mM imidazole pH 7.5) supplemented with DNAseI and EDTA-free cOmplete protease inhibitor cocktail (Roche) and lysed using a continuous flow cell disruptor (Constant Systems). Recombinant *Cp*/*Pf*KRS was purified via Ni^2+^ affinity chromatography followed by size exclusion chromatography. Purified *Cp*/*Pf*KRS was concentrated to ∼30 mg/mL in buffer (25 mM HEPES, 500 mM NaCl, 5% glycerol, 2 mM TCEP, pH 7.0), snap-frozen in liquid nitrogen, and stored at −80 °C.

### Crystallography and Crystallization

Crystals of *Cp*/*Pf*KRS were grown in similar conditions to those previously reported for *Cp*KRS.^13^ Protein (30 mg/mL) in storage buffer (25 mM HEPES, 0.5 M NaCl, 5% glycerol, 2 mM TCEP, pH 7.0) was incubated with 5 mM lysine prior to setting up crystallization drops. Crystals were grown using vapor diffusion in hanging drops, with reservoir containing 25% PEG 3350, 0.2 M lithium sulfate, and 0.1 M tris pH 7.8. Crystallization drops consisted of 1 μL of protein solution and 1 μL of reservoir. For soaking, crystals were transferred into drops consisting of 1 μL of reservoir and 1 μL of storage buffer containing 10 mM compound. Crystals were harvested after 1 h of soaking, cryo-protected using a reservoir supplemented with 33% glycerol, and flash-frozen in liquid nitrogen.

### Crystallography—Data Collection and Refinement

Data were collected at beamline I03 at Diamond Light Source at a wavelength of 0.97628 Å. The data were integrated using the DIALS automated pipeline^38^ and scaled and merged using Aimless.^39^ The structure was solved using the structure of *Cp*KRS (PDB: 5elo) as the search model in Phaser.^40^ Manual model building was performed using Coot,^41^ and the structure was refined using Refmac^42^ and incorporated into the CCP4 suite of software.^43^ The ligand dictionary was prepared using Grade (Smart et al., 2011), and model quality was assessed using Molprobity.^44^ Data collection and refinement statistics are given in Table S3.

## Supporting Information Available

Summary of primers used in qPCR and transgenic studies, summary of encoding SNPs identified in DDD01510706-resistant clones, X-ray crystallography data collection and refinement statistics, collated activity of DDD01510706 analogues in *Pf*KRS and *Hs*KRS enzymatic assays, identification of *P. falciparum* proteins that bind specifically to resin-bound linker 1 (low loading), identification of *P. falciparum* proteins that bind specifically to resin-bound linker 1 (high loading), TPP hits identified by *T*_m_ analysis of biological replicate 1, TPP hits identified by *T*_m_ analysis in biological replicate 2, NMR spectra of key synthetic chromone-based *Pf*KRS inhibitors, quantification of *Pf*KRS^WT^ and *Pf*KRS^S344L^ overexpression in transgenic lines by label-free quantitation and qRT-PCR, differential binding of *P. falciparum* lysate-derived proteins to “high load” resin-bound linker 1 in the presence of free DDD01510706 (100 μM) or DMSO, superimposed structures of *Cp*KRS:cladosporin, difference density (Fo-Fc) omit map for the ligand in the active site of chain A, contoured at the 2.5 σ level, with the final ligand coordinates superimposed, and TPP melt curves for *Pf*KRS following incubation with DDD0151076 (red) or vehicle (0.1% DMSO, black) (PDF)
