# Supplementary material for: Toolkit of Approaches To Support Target-Focused Drug Discovery for Plasmodium falciparum Lysyl tRNA Synthetase
Source: ACS Infect Dis. 2022 Aug 29;8(9):1962–74. doi: 10.1021/acsinfecdis.2c00364 (PMC9469095; doi:10.1021/acsinfecdis.2c00364)
Supplement: Supplementary file 1 — id2c00364_si_001.pdf [file id2c00364_si_001.pdf]

SUPPORTING INFORMATION

Toolkit of approaches to support target-focused drug discovery for *Plasmodium falciparum* lysyl tRNA synthetase

Rachel Milne, Natalie Wiedemar, Victoriano Corpas-Lopez, Eoin Moynihan, Richard J. Wall, Alice Dawson, David A. Robinson, Sharon M. Shepherd, Robert J. Smith, Irene Hallyburton, John M. Post, Karen Dowers, Leah S. Torrie, Ian H. Gilbert, Beatriz Baragaña, Stephen Patterson and Susan Wyllie[1]*

Wellcome Centre for Anti-Infectives Research, School of Life Sciences, University of Dundee, Dow Street, Dundee DD1 5EH, United Kingdom.

***Corresponding author:** Dr Susan Wyllie, Division of Biological Chemistry and Drug Discovery, Wellcome Centre for Anti-Infectives Research, School of Life Sciences, University of Dundee, Dow Street, Dundee DD1 5EH, United Kingdom.

Tel: (44)1382 38 5761; Email: s.wyllie@dundee.ac.uk

Supporting information

INDEX **Page**

Chemistry methods and details of synthesis **S3-6**

Supplementary tables

Table S1: Summary of primers used in qPCR and transgenic studies. **S7**

Table S2: Summary of encoding SNPs identified in DDD01510706-resistant clones. **S7**

Table S3: X-ray crystallography data collection and refinement statistics. **S8**

Table S4: Collated activity of DDD01510706 analogues in *Pf*KRS and *Hs*KRS enzymatic assays. **S9**

Table S5: Identification of *P. falciparum* proteins that bind specifically to resin-bound linker 1 (low loading). **S10**

Table S6: Identification of *P. falciparum* proteins that bind specifically to resin-bound linker 1 (high loading). **S11**

Table S7: TPP hits identified by T_m analysis of biological replicate 1. **S12**

Table S8: TPP hits identified by T_m analysis in biological replicate 2. **S12-13**

Supplementary figures

Figure S1: NMR spectra of key synthetic chromone-based *Pf*KRS inhibitors. **S14**

Figure S2: Quantification of *Pf*KRS^{WT} and *Pf*KRS^{S344L} overexpression in transgenic lines by label-free quantitation and qRT-PCR. **S15**

Figure S3: Differential binding of *P. falciparum* lysate-derived proteins to “high load” resin bound linker 1 in the presence of free DDD01510706 (100 µM) or DMSO. **S16**

Figure S4: Superimposed structures of *Cp*KRS:cladosporin. **S17**

Figure S5: The difference density (F_o-F_c) omit map for the ligand in the active site of chain A, contoured at the 2.5 σ level, with the final ligand coordinates superimposed. **S18**

Figure S6: TPP melt curves for *Pf*KRS following incubation with DDD0151076 (red) or vehicle (0.1% DMSO, black). **S19**

Chemistry general

Solvents and reagents were purchased from commercial suppliers and used without further purification. Dry solvents were purchased in sure sealed bottles stored over molecular sieves. 8-Amino-*N*-(cyclohexylmethyl)-6-fluoro-4-oxo-chromene-2-carboxamide[44] (**1**) was prepared under contract by WuXi AppTec by modification of a previously reported route. Air and moisture sensitive reactions were carried out under an inert atmosphere of nitrogen. Normal phase analytical thin-layer chromatography (TLC) was carried out on pre-coated silica plates (Kieselgel 60 F254, BDH) with visualisation via UV light (UV254/365 nm). Flash column chromatography was performed on a Combiflash Companion Rf (Teledyne ISCO) using pre-packed silica gel columns (230-400 mesh, 35-70 µm, Teledyne ISCO). ^{1}H NMR spectra were recorded on a Bruker Avance DPX 500 spectrometer (^{1}H at 500.1 MHz, ^{13}C at 125 MHz, ^{19}F at 470.5 MHz). Chemical shifts (δ) are expressed in ppm recorded using the residual solvent as the internal reference in all cases. Signal splitting patterns are described as singlet (s), doublet (d), triplet (t), quartet (q), multiplet (m), broad (br), or a combination thereof. Coupling constants (*J*) are quoted to the nearest 0.1 Hz. Low resolution electrospray (ES) mass spectra were recorded on an Advion Expression Compact Mass Spectrometer connected to a Thermo-Dionex Ultimate 3000 UPLC. High resolution mass spectra were recorded on a Bruker MicrOTOF II focus ESI Mass Spectrometer connected in parallel to Dionex Ultimate 3000 RSLC system with diode array detector. Compounds have been named using the ChemDraw Ultra 12.0 naming application which is commercially available from the CambridgeSoft Corporation. All stock solutions used in the synthesis of drug beads were prepared in high grade DMSO (Acros, 99.8%, for molecular biology). Drug beads were stored in high grade 2-propanol (iPrOH) (Sigma Aldrich, BioReagent, for molecular biology, >99.5%). Drug bead reactions were mixed using a Miltenyi Biotec MACSmix Tube Rotator.

Chemical synthesis

Synthesis of *N*-(cyclohexylmethyl)-6-fluoro-4-oxo-8-(phenylsulfonamido)-*4H*-chromene-2-carboxamide (**2**).

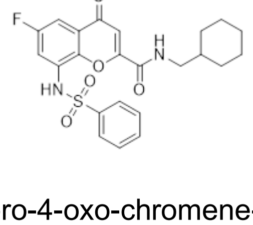

8-Amino-*N*-(cyclohexylmethyl)-6-fluoro-4-oxo-chromene-2-carboxamide (**1**) (0.124 g, 0.390 mmol) was added to a microwave vial, dissolved in anhydrous pyridine (2 mL) and cooled to

0 °C. Benzenesulfonyl chloride (0.08 mL, 0.635 mmol) was added dropwise, and the reaction mixture was allowed to warm to room temperature and stirred for 16 h. The reaction mixture was then diluted with EtOAc (10 mL), washed with 1 M aq. citric acid, dried over $MgSO_4$ and concentrated *in vacuo*. LCMS of the crude showed that the disulfonamide was the major species, therefore, the crude residue was dissolved in a solution of TBAF (1 M in THF, 1 mL) and stirred at room temperature for 1 h. The reaction mixture was poured into water (10 mL), extracted with EtOAc (3×10 mL), dried over $MgSO_4$ and concentrated *in vacuo*. The residue was dissolved in EtOAc (10 mL), passed through Celite® and concentrated *in vacuo* to give the desired product as a yellow solid (0.080 g, 40%). ^{1}H-NMR (500 MHz, MeOD): δ 7.80-7.67 (m, 3H, 3×ArH), 7.62-7.52 (m, 2H, 2×ArH), 7.51-7.42 (m, 2H, 2×ArH), 6.90 (s, 1H, CH), 3.30 (t, 2H, *J*=7.0 Hz, CH_2), 1.91-1.62 (m, 6H, 2× CH_2, CH, NH), 1.44-1.21 (m, 4H, 2×CH_2), 1.15-1.00 (m, 2H, CH_2) ppm. ^{13}C-NMR (125 MHz, MeOD): δ 177.2, 159.5, 159.3 (d, $^1J_{C-F}$ = 247 Hz), 155.5, 145.0, 139.3, 133.2, 128.9, 126.6, 125.3, 125.4, 117.0 (d, $^2J_{C-F}$ = 29 Hz), 110.3, 106.2 (d, $^2J_{C-F}$ = 24 Hz), 46.0, 37.8, 30.7, 26.1, 25.6 ppm. MS (ES+) *m/z*: 459 (100) $[M+H]^+$. HRMS (ES+): *m/z* calcd. for $C_{23}H_{24}FN_2O_5S$ $[M+H]^+$: 459.1390, found 459.1379 (-2.4 ppm).

Synthesis of 4-((2-(2-(2-(2-azidoethoxy)ethoxy)ethoxy)ethyl)carbamoyl)benzenesulfonyl chloride **(4)**

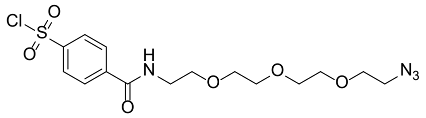

A suspension of 4-chlorosulfonylbenzoic acid (**3**) (0.175 g, 0.793 mmol) in anhydrous DCM (8 mL) was cooled to 0 °C and then oxalyl chloride (0.13 mL, 1.59 mmol) was added dropwise, followed by one drop of DMF (catalytic). The suspension was then allowed to warm to room temperature and stirred for 1 h. The mixture was concentrated *in vacuo* and then resuspended in anhydrous DCM (8 mL) and cooled to -78 °C. 1-Amino-11-azido-3,6,9-trioxaundecane (0.190 g, 0.873 mmol) was dissolved in a minimal amount of DCM and added dropwise to the reaction mixture, followed by DIPEA (0.14 mL, 1.06 mmol). The reaction mixture was allowed to warm to room temperature and then stirred for 20 h. The reaction mixture was then concentrated *in vacuo* and used immediately without purification.

Synthesis of 8-((4-((2-(2-(2-(2-azidoethoxy)ethoxy)ethoxy)ethyl)carbamoyl)phenyl)sulfonamido)-*N*-(cyclohexylmethyl)-6-fluoro-4-oxo-*4H*-chromene-2-carboxamide (**5**).

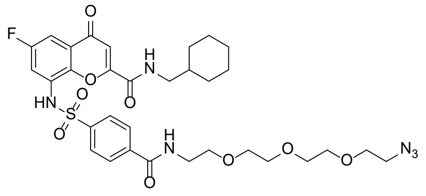

8-Amino-*N*-(cyclohexylmethyl)-6-fluoro-4-oxo-chromene-2-carboxamide (**1**) (0.176 g, 0.553 mmol) was added to a microwave vial and dissolved in anhydrous pyridine (2 mL). Crude 4-((2-(2-(2-(2-azidoethoxy)ethoxy)ethoxy)ethyl)carbamoyl)benzenesulfonyl chloride (**4**) dissolved in a minimal amount of ice-cold pyridine was added dropwise to the reaction mixture. The microwave vial was then capped and the reaction heated at 100 °C with stirring for 25 h. Upon cooling the resultant reaction mixture was diluted with EtOAc (10 mL), washed with 1 M aq. HCl, dried over $MgSO_4$ and concentrated *in vacuo*. Column chromatography (12 g silica, 100% EtOAc) gave the desired product as a yellow solid (0.073 g, 19%). ^{1}H-NMR (500 MHz, MeOD): δ 8.00-7.90 (m, 4H, 4×ArH), 7.80 (dd, 1H, *J*=9.5, 3.1 Hz, ArH), 7.63 (dd, 1H, *J*=7.9, 3.1 Hz, ArH), 6.99 (s, 1H, CH), 3.78-3.66 (m, 12H, 6×CH_2), 3.65-3.60 (m, 2H, CH_2), 3.46-3.35 (m, 4H, 3×NH, CH), 1.98-1.85 (m, 4H, 2×CH_2), 1.84-1.69 (m, 2H, CH_2), 1.49-1.30 (m, 4H, 2×CH_2), 1.20-1.07 (m, 2H, CH_2) ppm. ^{13}C-NMR (125 MHz, MeOD): δ 177.2, 167.0, 159.5, 159.9 (d, $^1J_{C-F}$ = 247 Hz), 155.6, 144.8, 141.9, 139.0, 127.9, 126.8, 125.4, 125.4, 116.5 (d, $^2J_{C-F}$ =29 Hz), 110.4, 106.1 (d, $^2J_{C-F}$ =24 Hz), 70.3, 70.2, 70.1, 69.9, 69.6, 69.0, 50.4, 46.0, 39.8, 37.8, 30.7, 26.1, 25.6 ppm. ^{19}F-NMR (470.5 MHz, MeOD): δ -114.16 ppm. MS (ES+) *m/z*: 703 (100) $[M+H]^+$. HRMS (ES+): *m/z* calcd. for $C_{32}H_{43}FN_7O_9S$ $[M+3H]^+$: 705.2715, found 705.2722 (+1.0 ppm).

Synthesis of 8-((4-((2-(2-(2-(2-aminoethoxy)ethoxy)ethoxy)ethyl)carbamoyl)phenyl)sulfonamido)-*N*-(cyclohexylmethyl)-6-fluoro-4-oxo-*4H*-chromene-2-carboxamide (**6**).

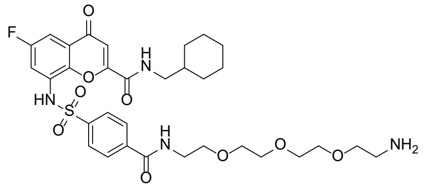

To a solution of 8-((4-((2-(2-(2-(2-azidoethoxy)ethoxy)ethoxy)ethyl)carbamoyl)phenyl)sulfonamido)-*N*-(cyclohexylmethyl)-6-fluoro-4-oxo-*4H*-chromene-2-carboxamide (**5**) (0.025 g, 0.0356 mmol) in a mixture of anhydrous MeOH (0.75 mL) and anhydrous THF (0.25 mL) at 0 °C was added nickel(II) chloride hexahydrate (0.0135 g, 0.057 mmol) followed by sodium borohydride (0.0062 g, 0.164 mmol). The reaction mixture was stirred at 0 °C for 4 h and then diluted with EtOAc (10 mL) and filtrated through Celite® which was subsequently flushed with MeOH. The filtrate was

concentrated *in vacuo*, resuspended in MeOH and purified using a 1 g SCX-2 cartridge to give the title compound as a yellow oil (16 mg, 60%). ^{1}H-NMR (500 MHz, MeOD): δ 8.01-7.96 (m, AA′BB′, 2H, 2×ArH), 7.88-7.84 (m, AA′BB′, 2H, 2×ArH), 7.35 (dd, 1H, *J*=11.5, 3.1 Hz, ArH), 7.02 (dd, 1H, *J*=8.2, 3.0 Hz, ArH), 6.99 (s, 1H, CH), 3.72-3.49 (m, 18H, 7×CH_2, CH, NH, NH_2), 3.03 (t, 2H, *J*=5.0 Hz, CH_2), 1.89-1.61 (m, 8H, 3×CH_2, 2×NH), 1.37-1.16 (m, 4H, 2×CH_2), 1.11-0.99 (m, 2H, CH_2) ppm. ^{13}C-NMR (125 MHz, MeOD): δ 179.2, 168.0, 160.2 (d, $^{1}J_{C\text{-}F}$ = 244 Hz), 160.1 156.5, 146.8, 146.3, 136.7, 127.3, 126.7, 124.9, 111.5 (d, $^{2}J_{C\text{-}F}$ =28 Hz), 109.7, 97.4 (d, $^{2}J_{C\text{-}F}$ =28 Hz), 70.1, 69.8, 69.7, 69.0, 66.5, 46.0, 39.5, 39.2, 37.8, 30.8, 26.2, 25.7 ppm. ^{19}F-NMR (470.5 MHz, MeOD): δ -115.80 ppm. MS (ES+) *m/z*: 677 (100) $[M+H]^+$. HRMS (ES+): *m/z* calcd. for $C_{32}H_{42}FN_4O_9S$ $[M+H]^+$: 677.2651, found 677.2619 (-4.7 ppm).

Supplementary tables

Table S1: Summary of primers used in qPCR and transgenic studies.

**Primers**	**Sequence (5' - 3')**
Pf KRS qPCR F (CDS 1607 - 1626)	ATTCGGCATTCTGCACTTCT
Pf KRS qPCR R (CDS 1725 - 1744, rev comp)	CTGGTCGCATAGTGGGAAAT
Pf β actin qPCR F	AAAGAAGCAGCAGGAATCCA
Pf β actin qPCR R	TGATGGTGCAAGGGTTGTAA
Pf AttB_P1	GAAAATATTATTACAAAGGGTGAGG
Pf AttB_P2	CTCTTCTACTCTTTCGAATTC
Pf AttB_P3	TTAGCTAATTCGCTTGTAAG
Pf LysRS _AttB_P4	CACTATGCGACCAGC

Table S2: Summary of encoding SNPs identified in DDD01510706-resistant clones.

Gene ID	Protein	Res 1	Res 2	Res 3
PF3D7_0809600	peptidase family C50, putative	-	P1460T	-
PF3D7_1149800	rifin	-	Y23X	Y23X
PF3D7_1350100	lysyl-tRNA ligase	S344L		
PF3D7_1428900	conserved Plasmodium protein	G130S	-	-

Table S3: X-ray crystallography data collection and refinement statistics.

**Data collection**	Values
Space group	$P2_1$
Cell dimensions	
a, b, *c* (Å)	73.10, 118.73, 143.45
α, β, γ (°)	90, 90.69, 90
Resolution (Å)	64.81-1.80 (1.83-1.80)
R_{merge}	0.0084 (0.991)
$I / \sigma I$	11.0 (1.7)
CC1/2	0.997 (0.840)
Completeness (%)	100 (99.6)
Redundancy	6.5, (7.0)
**Refinement**	Values
Resolution (Å)	1.80
No. reflections	226134
R_{work} / R_{free}	18.16/20.99
No. atoms	
Protein A/B/C/D	4034/4044/3960/4026
Ligand / lysine	49 / 10 (per chain)
Glycerol.	12/6/12/6
Water	1465
B-factors	
Protein A/B/C/D	33.8/33.3/34.0/36.2
Ligand A/B/C/D	42.8/47.3/51.6/46.9
Lysine A/B/C/D	22.3/23.8/22.8/27.6
Water	37.12
R.m.s. deviations	
Bond lengths (Å)	0.0092
Bond angles (°)	1.4523
PDB code	**7zog**

Table S4: Collated activity of DDD01510706 analogues in *Pf*KRS and *Hs*KRS enzymatic assays.

Compound	Enzymatic activity, pIC_{50}			
	*Pf*KRS	SD	*Hs*KRS	SD
DDD01510706	6.62	0.10	4.70	0.06
Cladosporin	7.32	0.10	<4	-
DDD02354914	7.93	0.05	5.2	0.1
Linker 1	7.65	0.2	4.36	0.1

Values represent the weighted mean ± SD of at least two independent biological replicates.

Table S5: Identification of *P. falciparum* proteins that bind specifically to resin-bound linker 1 (low loading). *P. falciparum* whole cell lysates were pre-incubated with either DDD01510706 (100 μM) or DMSO prior to incubation with compound-resin. Resin-bound proteins were analysed by LS-MS/MS with a high DMSO/compound ratio indicating specific binding to 13. Data shown for Log_2 DMSO/compound >1.5, 2.8-fold enrichment. Proteins shown were identified by the detection of at least 2 unique peptides.

Gene IDs	Log_2 ratio	Unique peptides	Protein description
PF3D7_1350100.1	7.28	32	lysyl-tRNA synthetase
PF3D7_0416300.1	2.04	3	DNA helicase MCM9, putative
P05109	1.98	2	Protein S100-A8; Protein S100-A8, N-terminally processed
PF3D7_1004000.1	1.92	10	60S ribosomal protein L13, putative
PF3D7_1124400.1	1.92	2	U6 snRNA-associated Sm-like protein LSm1, putative
PF3D7_1142600.1	1.92	5	60S ribosomal protein L35ae, putative
PF3D7_1462100.1	1.91	6	conserved *Plasmodium* protein, unknown function
PF3D7_1323100.1	1.90	4	60S ribosomal protein L6, putative
PF3D7_1323400.1	1.84	3	60S ribosomal protein L23
PF3D7_1027800.1; H7C422	1.78	15	60S ribosomal protein L3; RPL3 (human)
PF3D7_1338200.1	1.78	4	60S ribosomal protein L6, putative
PF3D7_1302800.1	1.70	4	40S ribosomal protein S7, putative

PF3D7_0503800.1	1.56	3	60S ribosomal protein L31
PF3D7_0714000.1	1.55	3	histone H2B variant
PF3D7_0517000.1; P30050	1.50	7	60S ribosomal protein L12, putative; RPL12 (human)

Table S6: Identification of *P. falciparum* proteins that bind specifically to resin-bound linker 1 (high loading). *P. falciparum* whole cell lysates were pre-incubated with either DDD01510706 (100 µM) or DMSO prior to incubation with compound-resin. Resin-bound proteins were analysed by LS-MS/MS with a high DMSO/compound ratio indicating specific binding to 4. Data shown for Log_2 DMSO/compound >1.5, 2.8-fold enrichment. Proteins shown were identified by the detection of at least 2 unique peptides.

Gene IDs	Log_2 ratio	Unique peptides	Protein description
PF3D7_1350100.1	4.70	32	lysyl-tRNA synthetase
P05109	4.40	2	S100-A8, *N*-terminally processed (human)
P06702	3.60	3	S100-A9 (human)
P04792; C9J3N8; F8WE04	1.58	2	heat shock protein beta-1 (human)

Table S7: TPP hits identified by T_m analysis of biological replicate 1.

Protein ID	ΔT_m 1	*p*-value	ΔT_m 2	*p*-value	Protein name
PF3D7_1350100	5.56	1.1E-06	5.60	5.3E-05	lysyl-tRNA synthetase
PF3D7_0905800	-2.52	2.0E-03	-4.50	2.1E-06	conserved *Plasmodium* protein, unknown function
PF3D7_1011000	4.23	2.9E-04	6.75	9.0E-07	inner membrane complex sub-compartment protein 1
PF3D7_1147300	3.26	1.7E-02	6.19	5.0E-16	conserved *Plasmodium* protein, unknown function
PF3D7_1443000	13.24	3.5E-26	6.81	7.4E-07	serine/threonine protein kinase
PF3D7_1451400	-4.18	2.1E-05	-4.29	4.5E-02	transcriptional regulatory protein sir2b
Q96IU4	-4.85	6.8E-03	-5.69	2.2E-02	putative protein-lysine deacylase (human)

Table S8: TPP hits identified by T_m analysis in biological replicate 2

Protein ID	ΔT_m 1	p-value	ΔT_m 2	p-value	Protein name
PF3D7_1350100	4.97	2.4E-12	4.55	3.4E-12	lysyl tRNA ligase
PF3D7_0108000	3.43	3.8E-08	3.61	3.7E-04	proteasome subunit beta type-3, putative
PF3D7_0519400	2.43	5.1E-04	2.45	7.2E-04	40S ribosomal protein S24

PF3D7_0821000	-2.46	1.9E-03	-3.19	3.0E-04	conserved *Plasmodium* protein, unknown function
PF3D7_1112400	-3.53	1.4E-07	-2.90	1.5E-03	nucleic acid binding protein, putative
PF3D7_1424100	3.26	5.3E-04	3.40	2.1E-07	60S ribosomal protein L5, putative
PF3D7_1436800	5.92	1.3E-03	4.21	4.5E-04	ATP-dependent Clp protease proteolytic subunit
PF3D7_1445500	6.03	2.9E-10	5.60	3.7E-04	conserved *Plasmodium* protein, unknown function
PF3D7_1464200	2.97	4.9E-06	5.07	7.0E-06	zinc finger CCCH domain-containing protein
Q96PZ7	-3.53	1.3E-07	-5.01	3.8E-21	CUB and sushi domain-containing protein 1 (human)

Supplementary figures

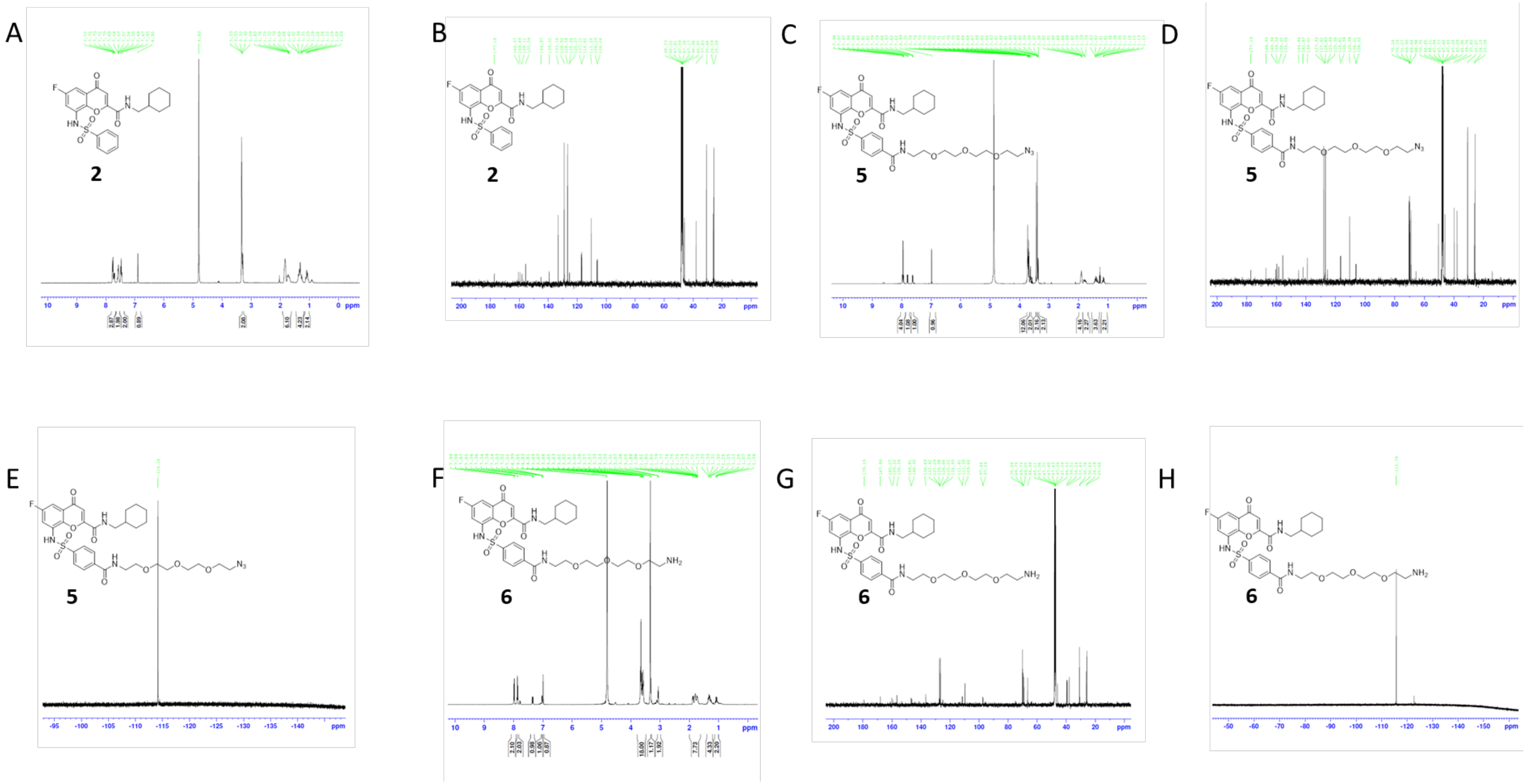

Figure S1: NMR spectra of key synthetic chromone-based *Pf*KRS inhibitors.

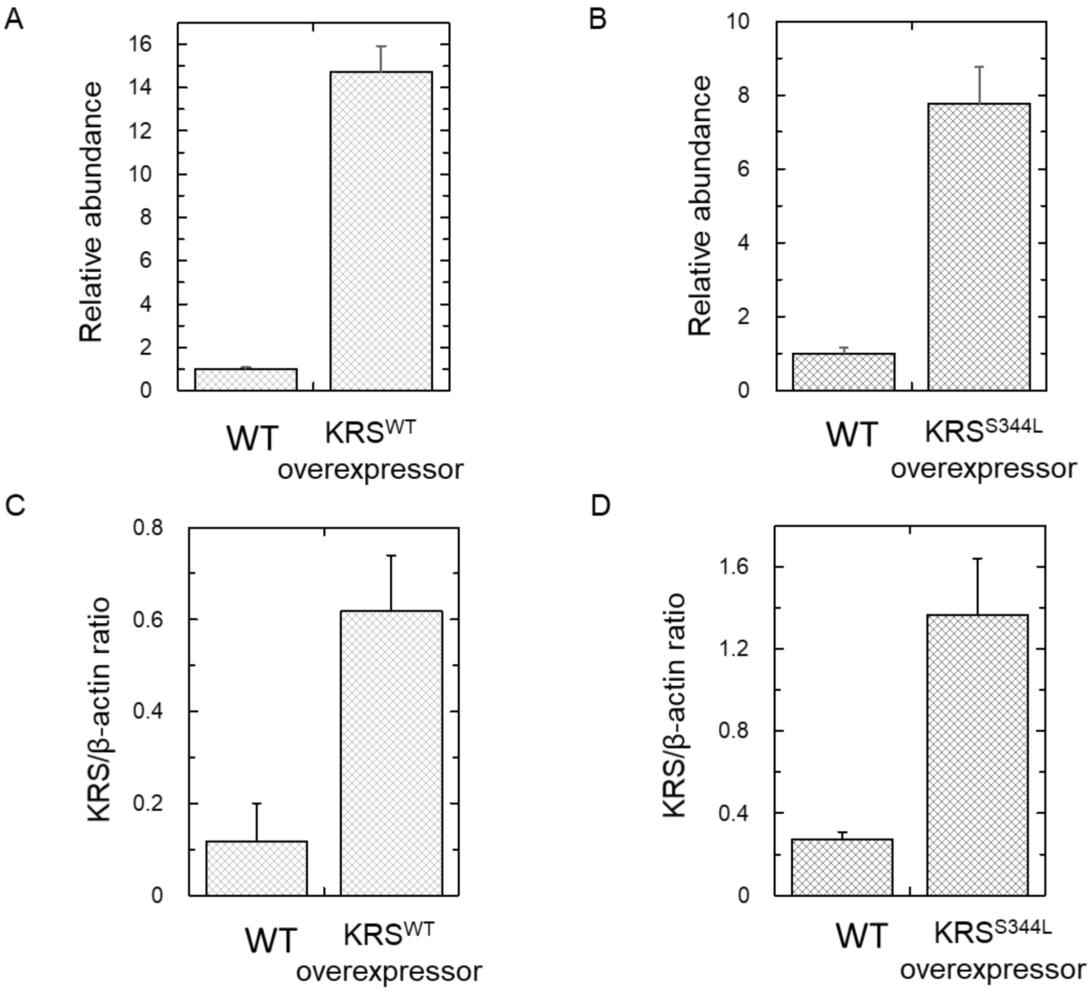

Figure S2: Quantification of *Pf*KRS^{WT} and *Pf*KRS^{S344L} overexpression in transgenic lines by label-free quantitation and qRT-PCR. Relative levels of transcript (A and B) and proteins (C and D) in WT and transgenic cell lines were directly compared. Details of these analyses can be found in the materials and methods.

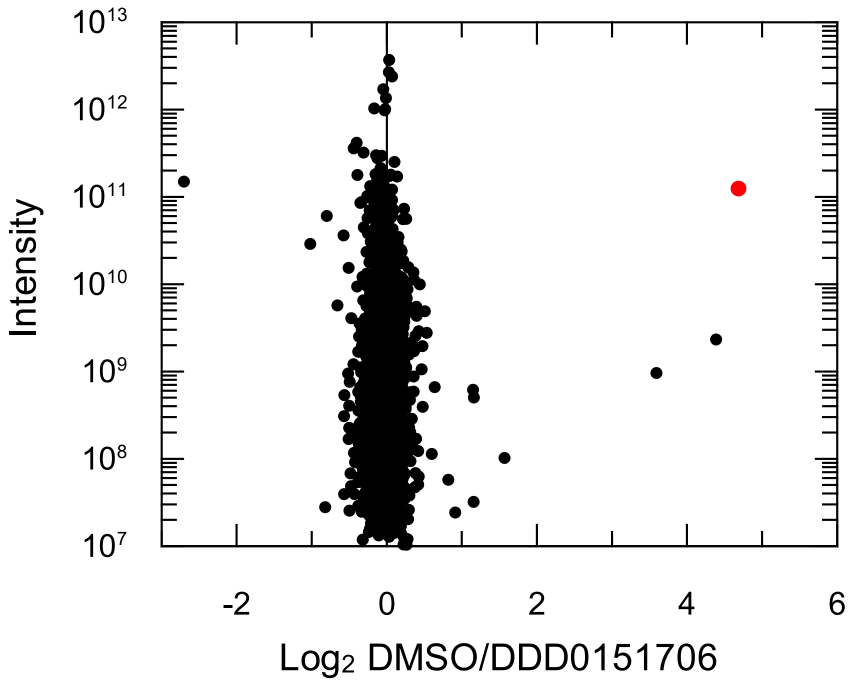

Figure S3: Differential binding of *P. falciparum* lysate-derived proteins to "high load" resin bound linker 1 in the presence of free DDD01510706 (100 μM) or DMSO. *Pf*KRS is highlighted in red.

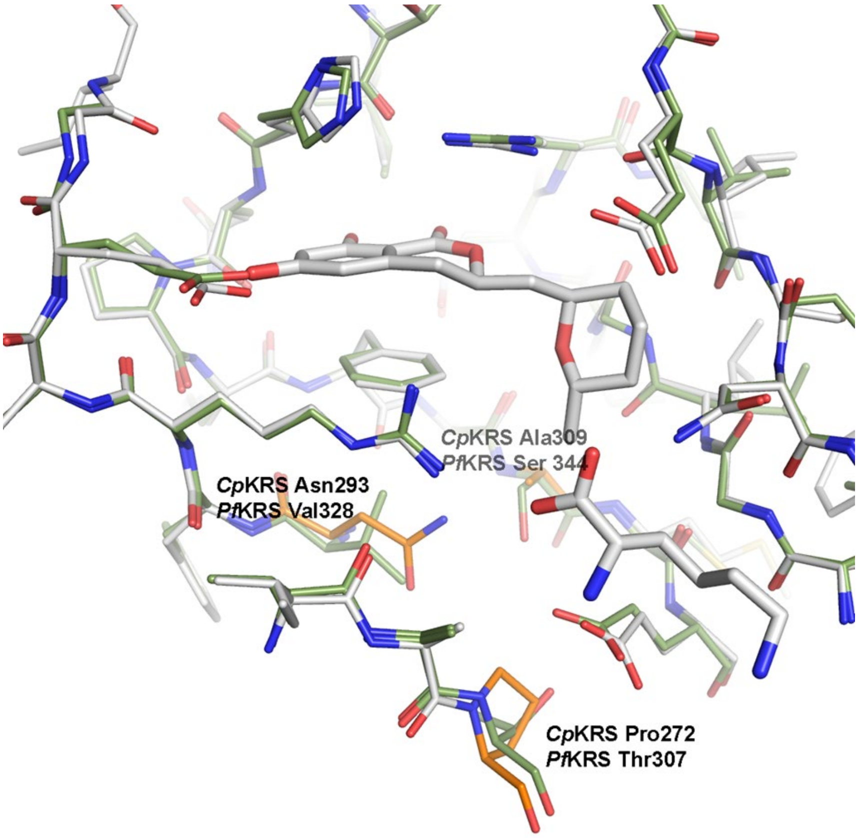

Figure S4: Superimposed structures of *Cp*KRS:cladosporin. PDB 5ELO, carbon atoms in grey) and *Pf*KRS (PDB 4PG3, carbon atoms in green)**.** *Cp*KRS residues selected for mutation in to mimic *Pf*KRS are shown with carbon atoms in gold.

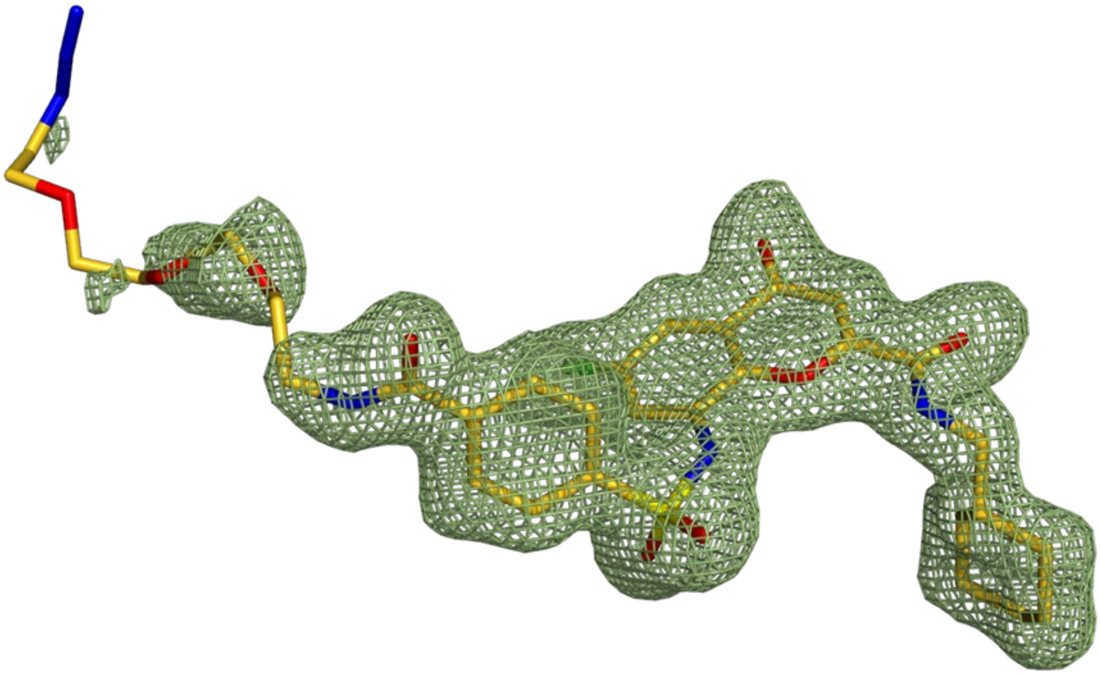

Figure S5: The difference density (F_o-F_c) omit map for the ligand in the active site of chain A, contoured at the 2.5 σ level, with the final ligand coordinates superimposed. The ligand is shown with carbon atoms in gold, nitrogen blue, oxygen red, sulfur yellow and fluorine in green.

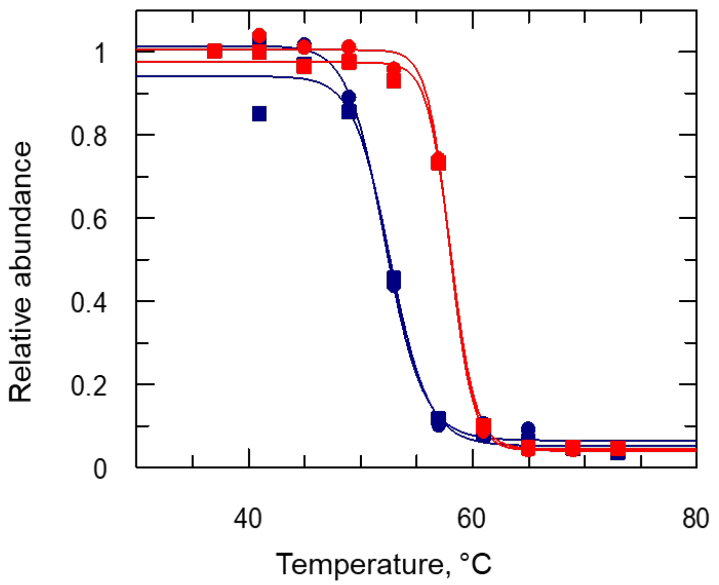

Figure S6: TPP melt curves for *Pf*KRS following incubation with DDD0151076 (red) or vehicle (0.1% DMSO, blue). Data from technical replicates (circles and squares) are shown, and the mean shift in melting temperature (ΔT_m) for *Pf*KRS in this biological replicate (replicate 2) was 4.8°C.
